# Archetypal transcriptional blocks underpin yeast gene regulation in response to changes in growth conditions

**DOI:** 10.1038/s41598-018-26170-5

**Published:** 2018-05-21

**Authors:** David Talavera, Christopher J. Kershaw, Joseph L. Costello, Lydia M. Castelli, William Rowe, Paul F. G. Sims, Mark P. Ashe, Chris M. Grant, Graham D. Pavitt, Simon J. Hubbard

**Affiliations:** 1 Division of Cardiovascular Sciences, School of Medical Sciences, Faculty of Biology, Medicine and Health, Manchester Academic Health Science Centre, The University of Manchester, Manchester, United Kingdom; 2 Division of Molecular and Cellular Function, School of Biological Sciences, Faculty of Biology, Medicine and Health, Manchester Academic Health Science Centre, The University of Manchester, Manchester, United Kingdom; 3 Division of Evolution and Genomic Sciences, School of Biological Sciences, Faculty of Biology, Medicine and Health, Manchester Academic Health Science Centre, The University of Manchester, Manchester, United Kingdom; 4 Manchester Institute of Biotechnology (MIB), The University of Manchester, Manchester, United Kingdom; 5 Present Address: Department of Biosciences, College of Life and Environmental Sciences, University of Exeter, Exeter, United Kingdom; 6 Present Address: Sheffield Institute for Translational Neuroscience, The University of Sheffield, Sheffield, United Kingdom; 7 Present Address: Department of Chemistry, Loughborough University, Loughborough, United Kingdom

## Abstract

The transcriptional responses of yeast cells to diverse stresses typically include gene activation and repression. Specific stress defense, citric acid cycle and oxidative phosphorylation genes are activated, whereas protein synthesis genes are coordinately repressed. This view was achieved from comparative transcriptomic experiments delineating sets of genes whose expression greatly changed with specific stresses. Less attention has been paid to the biological significance of 1) consistent, albeit modest, changes in RNA levels across multiple conditions, and 2) the global gene expression correlations observed when comparing numerous genome-wide studies. To address this, we performed a meta-analysis of 1379 microarray-based experiments in yeast, and identified 1388 blocks of RNAs whose expression changes correlate across multiple and diverse conditions. Many of these blocks represent sets of functionally-related RNAs that act in a coordinated fashion under normal and stress conditions, and map to global cell defense and growth responses. Subsequently, we used the blocks to analyze novel RNA-seq experiments, demonstrating their utility and confirming the conclusions drawn from the meta-analysis. Our results provide a new framework for understanding the biological significance of changes in gene expression: ‘archetypal’ transcriptional blocks that are regulated in a concerted fashion in response to external stimuli.

## Introduction

Cells are embedded in fluctuating environments, which pose physiological challenges. They must constantly balance the tradeoff between growth and defense against aggressive stimuli^1^. Challenges faced include infective agents, and internal and external stresses; e.g. oxidative stress, nutrient starvation, or exposure to radiation. Environmental stresses lead to concerted molecular responses in eukaryotic organisms that are driven by both transcriptional and post-transcriptional regulatory changes in gene expression and activity^1^. For instance, altered growth conditions influence the synthesis, turnover, location, and processing of particular RNAs^2–6^, as well as both global and individual protein synthesis rates, patterns of post-translational modifications and protein stability^7–9^.

Numerous studies have used *Saccharomyces cerevisiae* as a model eukaryotic organism for characterizing a broad range of stress responses at the transcriptional level, using DNA microarrays [e.g.^10–13^] and, more recently, next-generation sequencing (NGS) technologies [e.g.^14,15^]. These studies have defined many sets of genes that display coordinated expression changes in response to the varied environmental challenges. For example, experiments whereby cells grown aerobically in glucose-containing media were subjected to stress allowed the identification of the environmental stress response (ESR)^10,11^, which frequently involves the down-regulation of the RiBi regulon responsible for ribosome biogenesis^10–12^, and the up-regulation of genes involved in mitochondrial functions^10,13^. This common transcriptional response to stress may involve up to 15% of yeast genes^1^. Nevertheless, the regulation of stress-related expression changes is also considered to include multiple subtle condition-specific changes^1^.

Analyses of common DNA sequences upstream of co-regulated genes have facilitated understanding of the transcription factors (TFs) and their binding sites that contribute to the diverse controls. For example, stress-responsive TFs such as Msn2 and Msn4 have been shown to control the expression of many of the genes involved in ESR via the stress response element ‘STRE’^11,16,17^. Other TFs are also generally involved in multiple stress responses, in addition to more stress-specific fundamental roles; e.g. Hsf1 is a major regulator of heat shock responsive genes^18^, while Yap1 is key to the responses to oxidative stresses^19^, and Gcn4 activates transcription of amino acid biosynthetic genes following amino acid starvation^20^. Finally, other TFs play a role when yeast grows in optimal conditions, and they are inactivated under stress conditions; e.g. Mig1 and Mig2 repress the expression of numerous genes when glucose is available, hence glucose starvation leads to the transcription of their targets^21,22^. Thus, while there have been some attempts to rationalize the multitude of regulatory and signaling pathways controlling the stress response in yeast^23–25^, the transcriptional responses to each new environment are essentially unique^1^.

To study molecular stress responses, a common experimental paradigm involves the identification of differentially expressed genes that are either up- or down-regulated under a given stress; results can then be cross-compared with other studies. Although sensible, these approaches have some clear drawbacks and/or limitations. First, in many studies, cataloguing of activation or repression of gene expression is often done using arbitrary fold-change cutoffs (e.g. two-fold change) without ascertaining the statistical significance of changes. This could lead to biases based on transcript abundance, omitting relatively modest (but significant) changes in high abundance transcripts. Finally, there will be stochastic effects close to fold-changes cutoffs, including/excluding some genes by chance. Thus, we believe that the analysis of correlation of expression changes across a great number of experiments covering multiple stresses should be a more appropriate and powerful approach for studying the global mechanisms involved in the response to changing growth conditions^26^. By revisiting and interrogating the large body of differential gene expression data sets that are available in public repositories such as ArrayExpress^27^ and GEO^28^ we aim to address this challenge. We therefore performed a meta-analysis on 1379 stress-related microarray datasets deposited by numerous research groups, in order to discern general transcriptional principles of the environmental stress responses in yeast across a wide variety of stresses. We identified groups of RNAs that have similar patterns of variation across different stresses that we define here as Blocks of Transcriptional Responses (BTRs). Subsequent analyses of these BTRs demonstrated coherent, common functions suggesting that they operate as functional blocks, with common regulatory controllers, which are combined to generate stress-specific responses. Thus, this approach uncovered the relevance of various blocks of genes whose contribution is not dependent on their individual expression changes but their correlated variation. This suggests that responses to changes of growth conditions do not operate at the gene-level but, rather, at a block-level.

Finally, we evaluated and confirmed our findings with the analysis of a series of RNA-seq experiments. We examined the transcriptional changes in yeast following three different acute stress conditions: removal of glucose or amino acids, and addition of hydrogen peroxide to cause oxidative stress. These analyses demonstrate that these blocks are not circumscribed to microarray data, but are also evident in independent data sets produced by next generation sequencing. Our results delineate the intricate but coordinated stress-responses in yeast, and provide a novel functional framework for the analysis and interpretation of differential gene expression patterns in general that could be applied to other experimental systems.

## Methods

### Data for the meta-analysis

We used the SPELL database^26^ to identify datasets containing stress experiments. SPELL uses a controlled vocabulary that defines stress as “changes in the state or activity of yeast as a result of a treatment or mutation that results in stress and the associated stress response”^26^. In addition to this general term, datasets are also tagged with terms such as “chemical stimulus”, “heat shock” or “starvation”. Stress, as defined by SPELL, also contains mutations affecting specific genes, or treatments that could lead to untargeted genome modifications (e.g. “radiation”). We downloaded 86 double-channel microarray-generated datasets from the Saccharomyces Genome Database (SGD; http://www.yeastgenome.org) (see S1 File for information on the experiments). Those datasets presented the stress-caused relative variation (i.e. the log-ratio) for each RNA. We merged all datasets into a single matrix, which contained 6897 RNAs (mostly mRNAs, but also rRNAs, tRNAs, snRNAs and retrotransposons) and 1379 experiments (see S2 File). Most datasets did not contain replicates of the same stress conditions. When experimental replicates were available, we treated them as different experiments. Some cells in the matrix had missing values because (1) particular RNAs were not measured across all the experiments, or (2) the log-fold changes where listed as non-existent (likely to be treated as a reference experiment).

### Clustering of downloaded data

**Figure 1:**
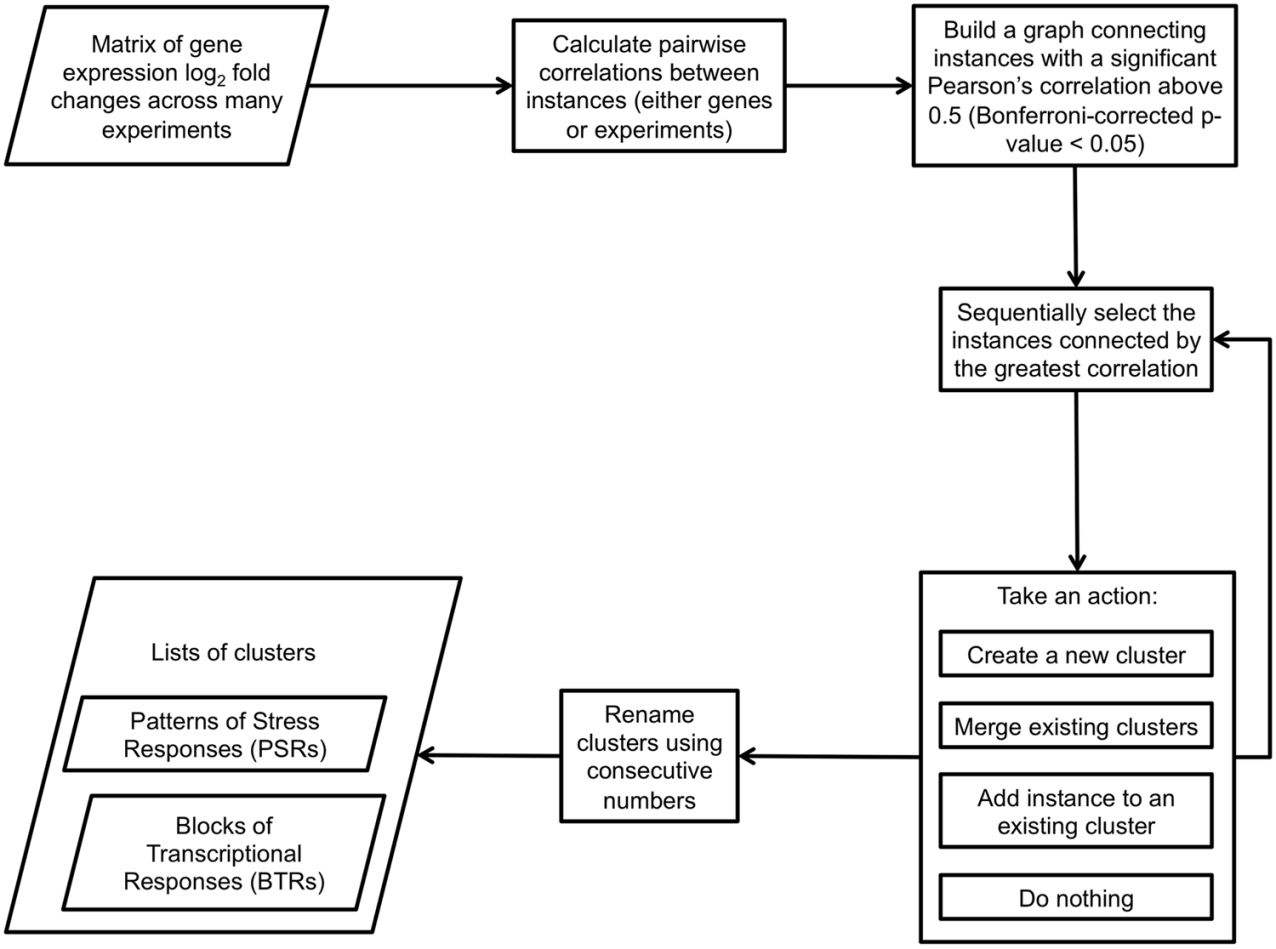
Flowchart of the clustering process. PSRs are clusters of arrays showing similar overall stress response. BTRs are clusters of RNAs that show a similar response to stress across multiple arrays.

We calculated the Pearson correlation coefficient between each experiment in the matrix (see S3 File). Then, we used an agglomerative clustering algorithm in order to group the experiments based on their correlations (i.e. the higher the correlation, the greater the similarity). We used a variation of the complete-linkage clustering method in order to cluster the experiments. Moreover, we required that all the elements in a cluster had a statistically significant correlation greater than 0.5. If this requirement was not met the experiment was assigned to a different cluster (see Figs 1 and S1 for an overview of the protocol). The resulting clusters were called Patterns of Stress Responses (PSRs). We used the same procedure in order to group the RNAs into Blocks of Transcriptional Responses (BTRs; see S4 File for the Pearson correlations between RNAs). In both cases, the numbering of the clusters only represents the order in which they were created. Information on the PSRs and BTRs is in the S1 File. The precise detail of identified clusters (number contents) depends on the clustering procedure used, which can be affected by the choice of both the linkage method, distance metric and the attendant threshold for membership. In order to investigate the robustness of our approach we explored the consistency of the generated PSR clusters by comparing our protocol to other approaches (see Figs S2–S5). Figure S2 shows that many of the clusters remain identical if using slightly different correlation thresholds, with the occasional expected merging or splitting of clusters when decreasing or increasing the threshold, respectively. Figs S3–S5 show how altering the linkage method does not result in extremely different clusters either, though minor, expected variations do occur. Another cause of concern was the possible existence of different biases between experiments; namely, a few data points driving the correlations up or down. In order to assess the effect of the actual distribution of log-fold changes in the clustering, we normalized the data using two quantile normalization approaches with the aroma.light package^29^. None of the normalization approaches show a dramatic effect on the clustering results (see Figs S6 and S7). More than half of the original clusters with 5 or more members are identical to clusters generated after rank-based quantile normalization. In the case of spline-based quantile normalization, 29% of clusters are identical and 58% have a Jaccard Index above 0.5. All alternative clustering results are presented in the S5 File.

### Identification of highly variable transcripts within PSRs

**Figure 2:**
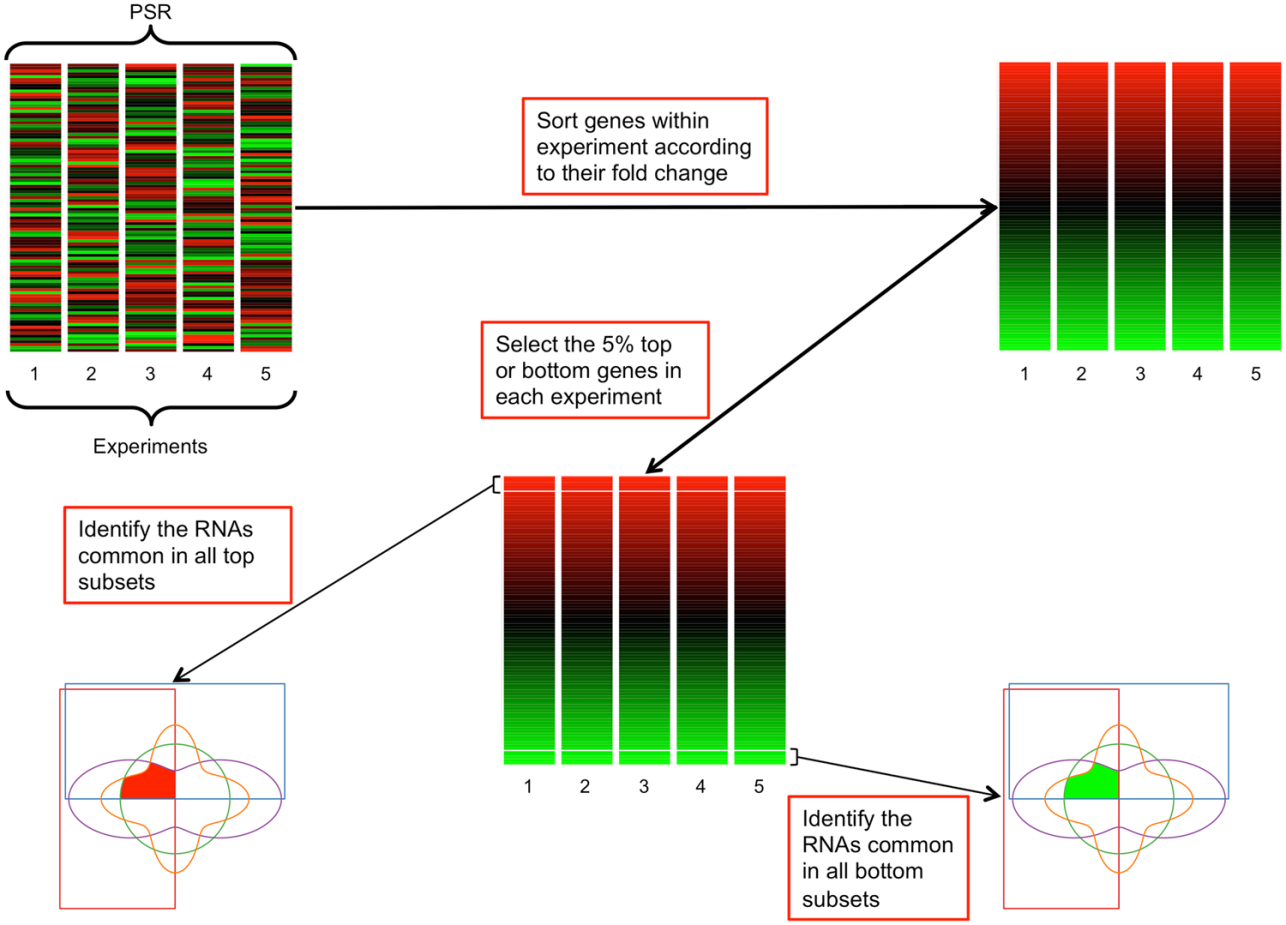
Flowchart for the identification of genes consistently enriched or depleted within a PSR. The flowchart shows the selection of common genes in the 5% top or bottom range of fold-changes. The same procedure was used for the 1% and 10% thresholds.

The variable origins of the expression data present difficulties in directly assigning significant gene enrichment or depletion within the context of each PSR; i.e. we considered it to be incorrect to use the same level of significance for the different datasets within a PSR. Therefore, we used an empirical alternative approach; namely, we selected RNAs that demonstrated a consistent response within the PSR (Fig. 2). For each experiment within a PSR, we ranked and identified the RNAs with the greatest log-fold change, identifying RNAs at three thresholds: 1%, 5%, and 10%. The RNAs found in common across the intersection of these percentile subsets (one for each experiment) were determined within the same PSR. These were then considered to be consistently over-expressed within the PSR. The same protocol was used in order to identify the RNAs that were consistently depleted, presumably down-regulated, within the PSR.

### Functional analyses

#### Involvement in Biological Processes

We used GO Slim for the functional annotation of Biological Processes (downloaded from the SGD on 3^rd^ April 2014). Yeast GO Slim is a cut-down version of the GO ontology, developed by the Saccharomyces Genome Database, which gives a general high-level description of the range of biological processes occurring in yeast; in keeping with our aims to detect enrichments in fundamental, common processes linked to general stress responses. Subsequently, we grouped the GO Slim Biological Processes into 15 broad categories (see S1 File for the recoding information). Fisher’s exact test was used in order to calculate the significance of association between the groups of genes and the GO Slim terms. The Benjamini-Hochberg procedure was used in order to correct p-values for multiple testing. Crucially, the use of GO Slim annotations greatly reduces the number of statistical tests to perform.

#### Physical and Genetic Interactions

Information on protein-protein interactions (PPIs) and genetic interactions (GIs) was obtained from the BioGRID (release 3.2.111; http://thebiogrid.org)^30^. We built the subnetworks of PPI and GI interactions for each BTR (we only took into account interactions linking two members of the same BTR). A random sampling procedure was used to test if the number of interactions was greater than expected by chance.

#### Transcription Regulation

Information on documented associations between genes and TFs was downloaded from Yeastract (http://www.yeastract.com)^31^. The Jaccard Index was used in order to analyse if RNAs in the same BTR or in different BTRs where controlled by common TFs (Fig. S8). Given the set of TFs controlling two RNAs, their Jaccard index is calculated as the ratio of common TFs over the total number of distinct TFs. We tested all pairwise comparisons within and between BTRs. When analysing common regulation within BTRs, we assigned to each BTR the median value of the within-BTR comparisons. Similarly, when analysing common regulation between BTRs, we assigned to each pair of BTRs the median value of their between-BTR comparisons. We used resampling in order to calculate the expected distributions of within-BTR and between-BTRs Jaccard indices. Finally, the association between BTRs and individual TFs was tested using Fisher’s exact test. P-values were corrected using the Bonferroni procedure in order to increase the stringency of this analysis.

### Analysis of expression of BTRs within PSRs

We analysed if individual BTRs as a group of genes were statistically overexpressed or depleted within each individual PSR. To minimize spurious results, we restricted this analysis to BTRs containing 10 RNAs or more, and to PSRs representing at least 5 experiments. For each BTR-PSR analysis we selected the expression values corresponding to the subset of RNAs within those particular experiments. Then we calculated the median expression value for each RNA across the experiments contained with that PSR. We used that vector of median expression to test if the distribution of values was spread along largely positive or negative fold changes (Wilcoxon signed rank test for a single sample, which effectively tests if the median of the vector is located at 0). As many such tests were performed we only considered significant those with an FDR-corrected p-value below 0.05. The median value of the vector was used in order to identify the sign of the bias (either enrichment or depletion).

### Yeast growth conditions

BY4741 *HIS3*^+^ strains^32^, bearing a genomically-integrated tandem affinity purification tag attached 3′ and in frame with a different translation initiation factor (eIF4E, eIF4G1 or eIF4G2), were grown to an OD_600_ 0.6 in SCD –His (single drop-out Kaiser mix, Formedium Ltd). We have previously shown that each TAP-tag does not impact on protein expression levels of the tagged partner, or PPIs among these factors or cell growth, and overall translation^32^. This culture was then split and exposed to amino acid starvation, glucose starvation, or oxidative stress, following previous protocols^33–37^. An unstressed control was also processed from the same culture. For amino acid starvation, the culture was centrifuged and resuspended in pre-warmed SD lacking all amino acids, this was repeated to wash the cells and the resulting culture was incubated for 15 mins. For glucose starvation and the control, the culture was resuspended in pre-warmed SC–His or SCD-His and incubated for 10 mins. Oxidative stress was achieved by adding hydrogen peroxide to a final concentration of 0.4 mM and incubating for 15 mins. After the stated incubation time each culture was centrifuged and resuspended in 50 ml of pre-warmed SCD–His containing 3% D and 2x amino acids (control), SD with 3% D (amino acid starvation), SC containing 2x amino acids (glucose starvation) or SCD with 0.4 mM hydrogen peroxide containing 3% D and 2x amino acids (oxidative stress) and centrifuged. Cell pellets were snap frozen in liquid nitrogen and subsequently ground into Buffer A^32^.

### RNA extraction, NGS and bioinformatics post-processing of data

RNA extraction and sequencing library generation was performed as described previously^32^. Nine biological-replicate samples for each stress were sequenced on an ABI SOLiD^®^ sequencer: these nine replicates consisted of three replicates each of three different BY4741-derived ‘wild-type’ strains. Bioinformatics post-processing of sequencing reads was performed as described previously^32^. Raw files of reads are deposited in ArrayExpress (E-MTAB-5836). Control experiments are also deposited in ArrayExpress (E-MTAB-2464).

### Differential gene expression analyses

Differential gene expression analyses were performed with the edgeR package^38^, using the generalized linear model (GLM) approach to test for significant changes in gene expression due to the diverse stress whilst also controlling the effect of having different tags. We used FDR < 1% in order to select significantly enriched or depleted transcripts. The average amount of transcripts (RPKM) was calculated by subtracting the base-2 logarithm of the gene length in kilobases from the average log_2_CPM estimated by edgeR.

### Multiple testing corrections

In most of the analyses performed in this work, we used the Benjamini-Hochberg correction for multiple testing. This correction procedure aims to control the rate of False Discoveries; i.e. which proportion of tests that we consider statistically significant are indeed false positives. Therefore the use of a particular FDR threshold reflects the willingness to accept fewer or more False Discoveries. We used different thresholds within the study depending on the analysis undertaken, and the statistical power; the greater the statistical power, the stricter the threshold.

We used the Bonferroni correction when willing to use an overtly conservative approach: (1) when clustering the data, and (2) when building the network of Transcription Factors associated with BTRs. This ensured that we only focused our discussion on very likely true associations.

### Data availability

NGS datasets analysed in this study are publicly available in ArrayExpress (E-MTAB-5836 and E-MTAB-2464). Supplementary Files published along this paper contain the microarray datasets assembled for this study as well as the results described/discussed here.

## Results

### Transcript depletion plays a major role in cell responses to stress

To generate a comprehensive experimental data set of transcriptional responses to changes in growth conditions we collated 1379 published microarray datasets from the SPELL database^26^. We restricted our analyses to datasets containing stress experiments that reported log-ratio stress-response results (see Methods for details). To define consistent sets of stress responses, we calculated pairwise Pearson correlation coefficients between all the experiments, which were subsequently partitioned into clusters (all experiments within a cluster must have a correlation above 0.5; see Figs 1 and S1 for a summary of the protocol). These clusters of experiments display coherent Patterns of Stress Response, which we term PSRs. In total, we found 216 PSRs (containing between 2 and 79 experiments each; Fig. S9 and S1 File), and 152 singleton experiments. Although some PSRs had a mixed structure, 87% of the PSRs were coherent single-study experiments; i.e. all the experiments in the PSR had been published in a single article. This can be explained by: (1) some of the smallest PSRs only contain repeats of the same experiments, (2) the stresses tested in any one study were generally very similar to each other, and/or (3) the experimental set-up could have greater influence than the stress itself^39–41^. To illustrate the latter point, PSR 100 contains 79 datasets analysed using *in-situ* Affymetrix arrays, and PSR 55 contains 37 datasets analysed using non-commercial spotted arrays. This effect was also supported using alternative clustering approaches, where some datasets in PSR 100 were additionally linked to those in PSR 32, which also contains datasets analyzed using Affymetrix arrays (see Figs S2–S7). Similarly, alternative clustering strategies led to the merging of other non-commercial spotted arrays datasets with PSR 55. To identify archetypal transcriptional responses associated with a coherent stress response cluster, we limited further analysis to 74 PSRs comprising 5 or more experiments (S6 and S7 Files). The majority of these PSRs contain single time course experiments, but 4 include experiments from multiple studies.

For each single experiment in a PSR we identified the most up-regulated RNAs, identifying the top 10, 5 or 1% rather than using fold-change cut-offs which are likely to vary between experiments. These three stringencies represent alternative but internally consistent approaches to defining contributors to the stress-response. For example 10% is a lenient threshold as it assumes that around 20% of genes should be involved in the stress-response (doubling the ~300 induced ESR genes suggested by previous analyses^1^). After identifying those up-regulated genes in each experiment, we identified the intersection of these gene subsets within the PSR in order to identify those RNAs that were consistently enriched across the whole PSR (Fig. 2 for a summary of the approach). We also repeated the same process with the most repressed RNAs (bottom 10, 5 or 1% RNAs). We found that the extent of response consistency was PSR-specific. For instance, selection of top and bottom 5% of RNAs resulted in a moderate anti-correlation between the number of experiments in the PSR and the combined size of the intersected sets (ρ = −0.43, p-value < 0.01). However, there was no correlation between the size of the up-regulated and depleted gene sets (ρ = 0.14, p-value ≈ 0.24). Hence, whereas the number of up and down-regulated genes for each experiment was broadly the same, the size of the common regulated up and down gene sets for each PSR differ markedly (Figs S10–S12). The Wilcoxon paired tests show that there are no trends in regards with whether common up-regulated or down-regulated genes are more numerous (p-values > 0.05). In other words, some stress PSRs involve down-regulation of a wider set of genes than are enriched, while for other PSRs the reverse is true.

**Figure 3:**
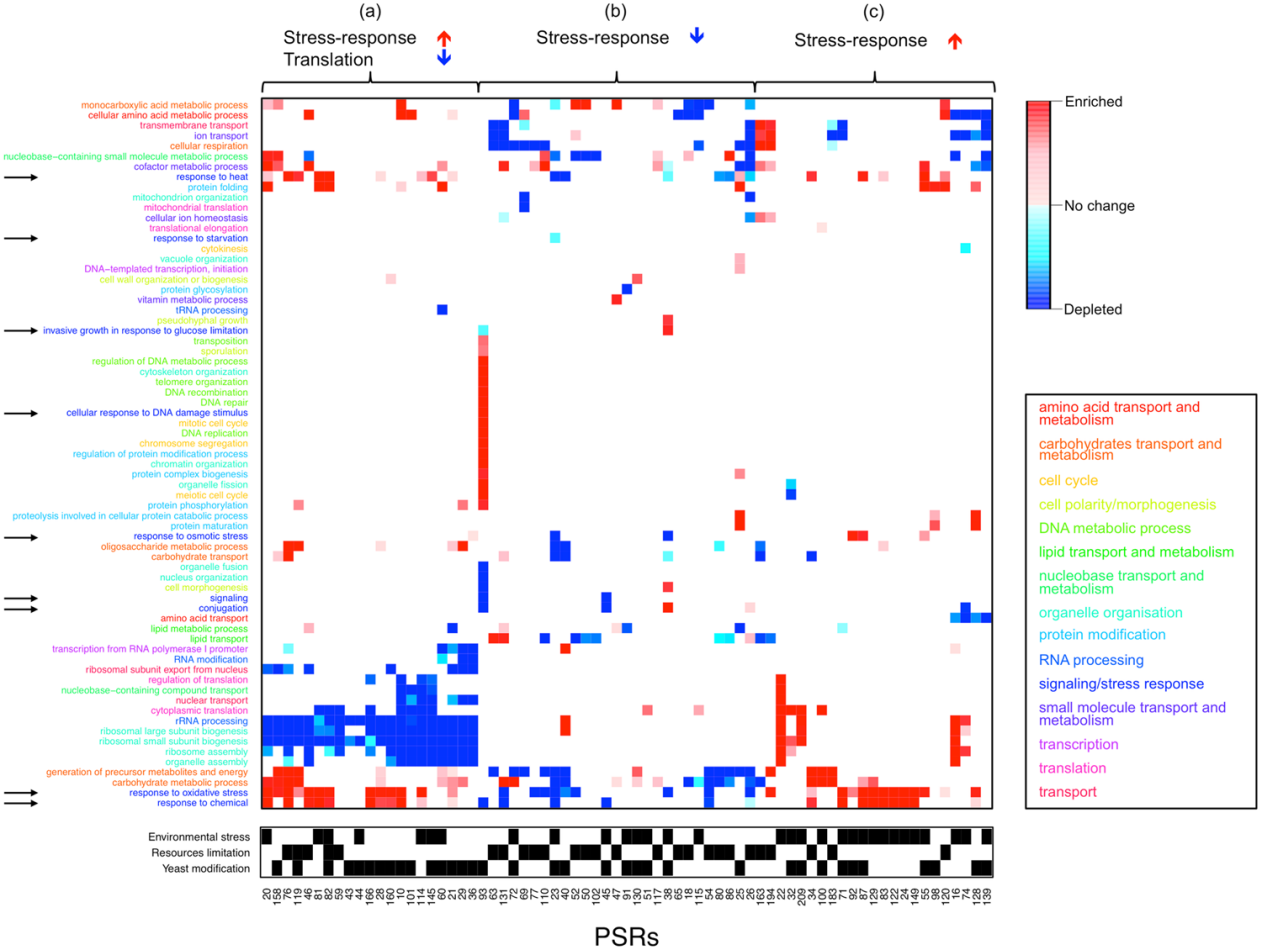
Biological processes affected by consistent stress response (top/bottom 5% RNAs). GO Slim terms overrepresented in the set of 5% most-enriched transcripts (red) or the set of 5% most-depleted transcripts (blue). The darker the colour the more statistically significant is the over-representation (non-significant biological processes are shown in white). Terms are grouped in 15 categories, and their name coloured accordingly. Signaling/Stress response biological processes are marked with a black arrow. The black squares refer to a generalized type of stress observed in individual PSRs. The order of both PSRs and biological processes depends on the hierarchical clustering of Euclidean distances calculated along the columns and rows respectively.

We studied if the sets of genes showing the greatest consistent expression changes were statistically associated with specific GO Slim Biological Process terms. Our functional enrichment analyses proved that those sets of coherently induced/repressed genes were associated with particular biological processes (see Figs 3, S13 and S14, and S8 File). As expected from stress-response datasets many PSRs contained up-regulated transcripts of genes related to stress-responses (arrowed in Fig. 3). PSRs that activate the stress response could be split into two broad subgroups: those that also shut down ribosome biogenesis (group a) and those that did not (group c). This highlights differences in the necessity of saving energy by stopping ribosome synthesis. Additionally, PSRs from experiments involving changes in resources availability (i.e. affecting the levels of nutrients or oxygen) showed a repression of many stress-response gene classes (group b). It is likely that many of those experimental set ups are compatible with active growth; e.g. recovery from stress.

Although several previous studies observed stress-induced activation of multiple genes with mitochondrial functions^10,13^, notably, we observe few PSRs where mitochondrial biological processes (such as cellular respiration, mitochondrial organization and mitochondrial translation) are overrepresented. This could be due to several reasons: (1) many mitochondrial proteins participate in broader biological processes such as carbohydrate metabolism or generation of energy; (2) mitochondrial functions are only important in specific stresses; and, (3) some mitochondrial functions might be fulfilled by diverse proteins, resulting in a lack of common genes within PSRs. Looking at those PSRs that show an effect in mitochondrial biological processes, we found that in some cases there was an inhibition of cellular respiration that accompanied the depletion of stress-response genes (especially those related to oxidative stress). Conversely, the activation of respiration might be related to a stress-response without translation inhibition.

Summing up, the PSRs constitute three broad coordinated responses shared across a wide portfolio of cell stresses and changing growth conditions: (1) upregulation of stress response mRNAs accompanied by the depletion of ribosome synthesis; (2) depletion of stress (and possibly cellular respiration) RNAs; and, (3) induction of stress response (and sometimes cell respiration) RNAs without significantly altering ribosome synthesis. These three global stress responses are essentially independent of the cutoff used to define common gene sets, when considering the top/bottom 5% or 10% of RNAs, but they are not apparent when selecting only the top/bottom 1%; the latter are the RNAs with the greatest fold change variation. For example, although ribosome biogenesis and cytoplasmic translation are part of the global response (Figs 3 and S13), few PSRs filtered by the 1% of top/bottom differentially expressed genes are involved in these biological processes (Fig. S14); only the inhibition of TOR by rapamycin (PSR 10) causes a massive depletion of those transcripts.

### BTRs are coherent functional blocks of RNAs

In order to explore the relevance of coordinated variation we observed in PSRs we performed a second complementary analysis of the same transcriptomic stress datasets. Here we grouped individual RNAs (rather than the experiment groups analysed above) based on the Pearson correlation between their expression changes across all 1379 experiments defined previously (Figs 1 and S1). RNAs were assigned exclusively to a single group, which we termed Blocks of Transcriptional Responses (BTRs). We found 1388 BTRs, each containing between 2 and 148 RNAs that all have coherent expression with Pearson correlations >0.5 (Fig. S15 and S1 File). In total, only 984 RNAs out of 6897 were not assigned to any BTR.

We studied the reasons for such a coordinated transcriptional response despite the diversity of the individual experiments. Previous studies had noted such co-expression, for example between components of protein complexes^42^, signaling pathways^43,44^, and metabolic pathways^44^. Our analyses showed that at least 13% of BTRs contained functionally-related RNAs; i.e., there was a statistically significant association between the RNAs in particular BTR and some GO Slim biological processes (see Figs S16, S17 and S18A, and S9 and S10 Files). Individual BTRs can be associated with many biological processes, and different BTRs can be associated with the same biological process. For example, BTR 318, BTR 381 and BTR 856 are involved in several processes related to protein modification, whilst BTR 183, BTR 192, BTR 209, BTR 276, BTR 380 and BTR 763 are all involved in the same processes of carbohydrate metabolism (Fig. S17). We also noted that members of individual BTRs had more protein-protein interactions (PPIs) and genetic interactions (GIs) than expected by chance (Figs S18 and S19**)**. Both types of interaction can be used as a proxy for functional similarity: proteins interact when participating in the same biological processes, and there is a clear link between gene function and epistasis^45–47^. PPIs can be obligate protein complexes or transient interactions (such as those involved in signal transduction or metabolic pathways). GIs occur when double mutants cause unexpected phenotypic consequences. Previous analyses have related ‘positive’ interactions to membership of protein complexes^48^, and ‘negative’ GIs to backup or redundant pathways^49^. In sum, across all these sources of evidence, 19% of BTRs are enriched for common function, pointing towards a generic stress-linked role for these BTRs. Functionally-coherent BTRs tend to contain multiple transcripts; e.g. 41% of them contain 5 or more transcripts (see Fig. S18D). It is important to recall that the BTRs do not represent singular responses to one environmental condition, but a common multi-stress coherent gene expression response.

**Figure 4:**
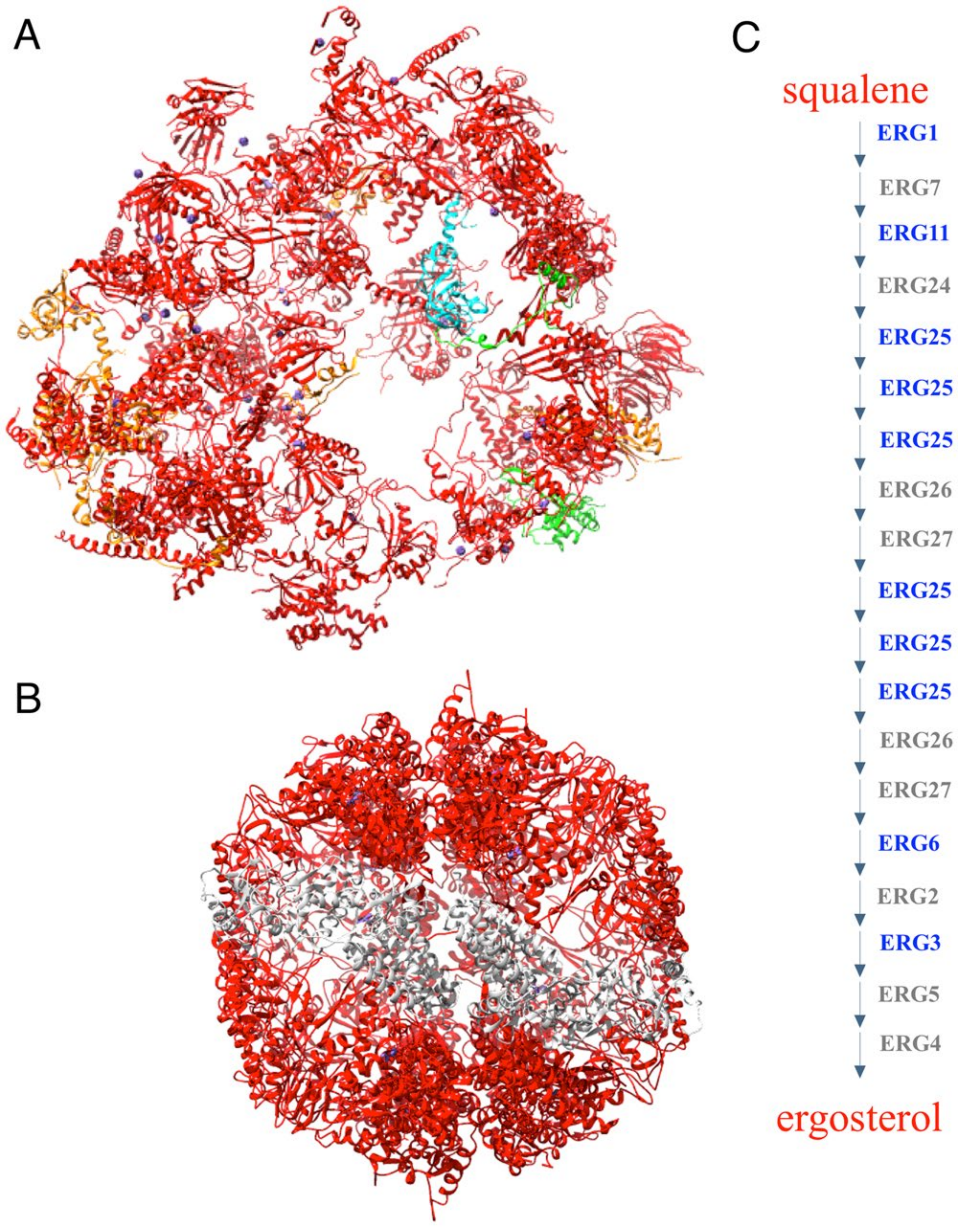
Functional coherence of BTRs. (**A**) 80S ribosome (PDB:4u52); BTR 180, red; other colours represent proteins whose genes are included in other BTRs. (**B**) Chaperonin complex (PDB:4v8r); BTR 224, red; Cct6, in grey. (**C**) Ergosterol synthesis pathway; transcripts in BTR 207 are shown in blue.

**Figure 5:**
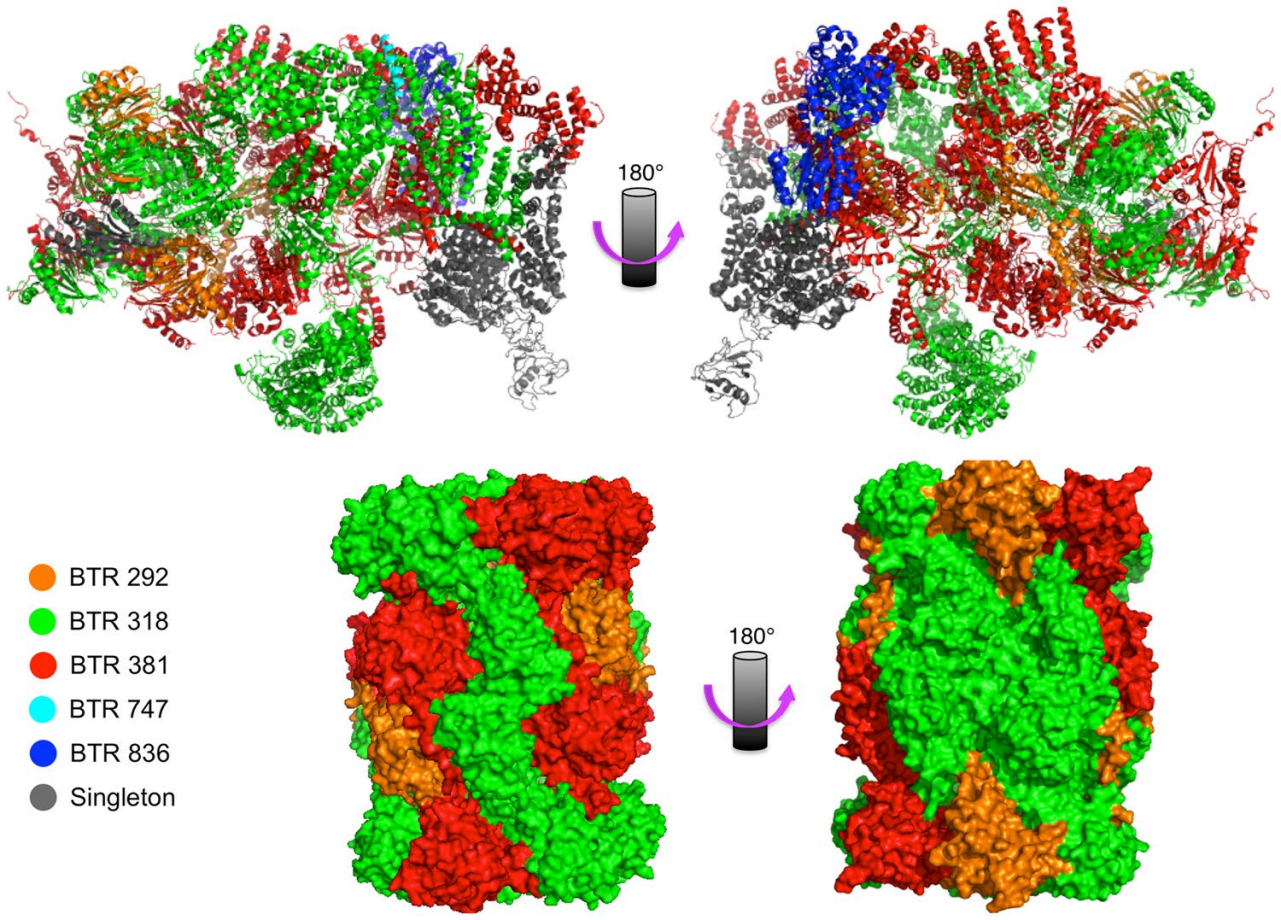
BTRs within proteasome. Front and back views of the 26S (top; PDB:4cr4) and 20S (bottom; PDB:5cgf) proteasomes.

Inspection of these functionally-coherent BTRs demonstrated several with a significant overlap with the protein machinery responsible for the synthesis, folding and degradation of proteins. For example, BTR 180 is one of the largest BTRs and contains the majority of cytoplasmic ribosomal proteins (similar to the RP regulon), as well as several translation initiation and elongation factors. Ribosomal proteins are present in other BTRs (e.g. BTRs 173 and 235); however, many of them are paralogues of transcripts included in BTR 180. Other examples of BTRs representing protein molecular machines include BTR 224, which contains 7 out of 8 proteins of the chaperonin complex (*CCT6* was not included in any BTR); and, BTRs 318 and 381, which contain most of the 20S core subunit of the proteasome and many regulatory proteins necessary to assemble the functional 26S proteasome (Figs 4A,B and 5). Other BTRs overlapped with the molecular machinery involved in energy production: BTR 209 contains many proteins forming the cytochrome c oxidase, whereas BTR 258 contains many members of the ATP synthase complex. Hence this unbiased clustering of transcriptome data across diverse conditions points to common “hallmarks” of co-regulation of macromolecular complexes in response to stress in *S. cerevisae*.

Nevertheless, not all BTRs correspond to molecular machines; several relate to metabolic pathways. For example, BTR 207 is involved in the ergosterol biosynthesis pathway^50–53^, containing 5 of the 12 mRNAs encoding enzymes necessary for transforming squalene into ergosterol: Erg1, Erg3, Erg6, Erg11, and Erg25 (see Fig. 4C). Erg1, Erg11 and Erg25 participate in redox reactions with paired donors (E.C. 1.14). Additionally, BTR 207 also contains *NCP1*, which encodes a NADP-cytochrome P450 reductase associated with Erg11^54^. Other components of this metabolic pathway co-occur in other BTRs; e.g., *ERG7* and *ERG26* are co-expressed, as are *ERG24* and *ERG28*, which encodes an endoplasmic reticulum membrane protein that interacts with Erg26 and Erg27. In regards to *ERG27*, this gene is co-expressed with *ERG8*, *ERG12* and *ERG13* (members of the mevalonate pathway), *ERG20* (enzyme that links the mevalonate and ergosterol pathways), and *ACS2*, which is one of two genes that transform acetate into acetyl-CoA. These 6 genes form BTR 490.

Further examples include BTRs 102, 156 and 201. BTR 102 includes *GAL1*, *GAL7* and *GAL10*, responsible for transforming β-D-galactose into glucose-1-phosphate^55–57^, as well as the galactose permease *GAL2*^58^. BTR 156 contains the four copies of *ASP3*, whose expression are induced during nitrogen starvation, and are responsible for the degradation of L-asparagine to L-aspartate^59,60^. BTR 201 contains *MET3, MET5, MET10, MET14* and *MET16*, which are involved in the sulfate assimilation pathway^61^. It also contains *MET17*, encoding the enzyme which uses hydrogen-sulphide to transform O-acetyl-L-serine into homocysteine^61^, and *MXR1*. Mxr1 protects iron-sulfur clusters from oxidation during oxidative stress^62^. Hence BTRs represent coherent functional blocks of RNAs that are coordinately expressed across diverse stress conditions. Such coordinated responses would help maintain stoichiometry of protein complexes, and/or metabolic pathways.

### BTR mRNAs are co-regulated by specific TFs

**Figure 6:**
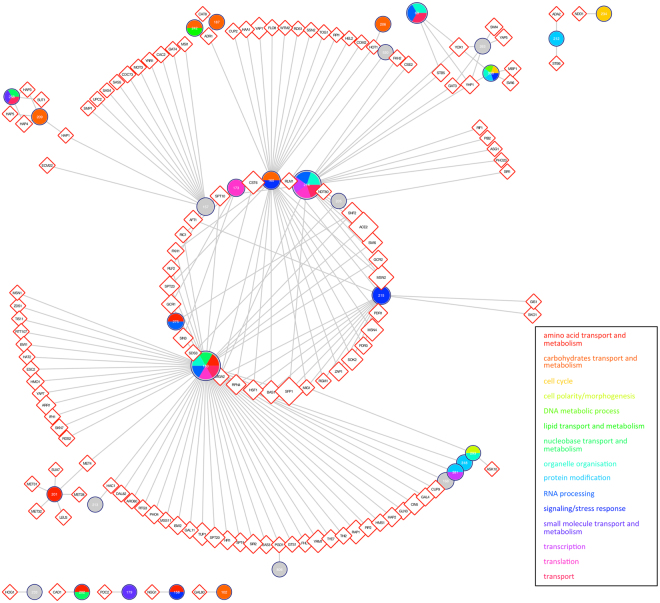
Network of transcription factors associated with BTRs. We only included associations with Bonferroni-corrected p-value < 0.01. Circles represent BTR. Their diameter is proportional to the size of the BTR, and their colours show the overrepresented Biological Processes within. Diamonds are transcription factors, and their size is proportional to the number of their target genes.

**Figure 7:**
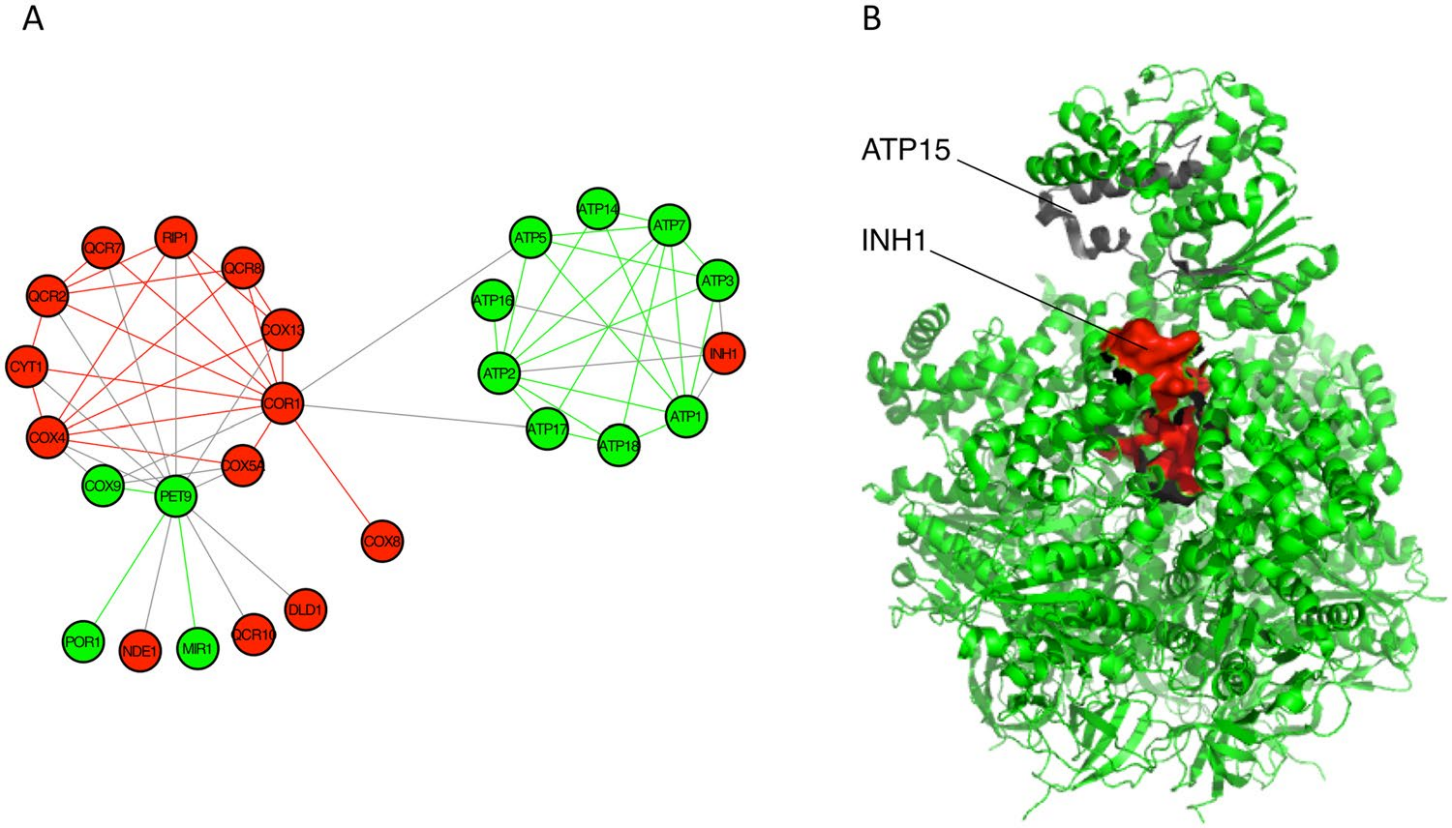
BTRs in oxidative phosphorylation pathway. (**A**) Network of physical interactions between members of BTRs 209 (red nodes) and 258 (green nodes). Red and green lines (edges) show within-BTR interactions, whereas between-BTR interactions are coloured grey. (**B**) F1-ATPase (green and grey) complexed with Inh1 (red) (PDB:3zia). F1-ATPase is coloured in green (BTR 258) and grey (Atp15, which is clustered with *ATP4* in BTR 915). *INH1* is a member of BTR 209.

The modular nature of the regulatory network has been frequently used for rationalizing gene expression changes^44,63^; specific TFs (and co-regulators) are associated with coordinating the co-expression of genes involved in particular biological processes. We reasoned that such relationships might also be true for BTRs. According to Yeastract data^31^, the vast majority of genes (99.3%) are controlled by more than one TF. Correspondingly, we found that the expression of genes within individual BTRs is coordinated by a set of common TFs, which are non-randomly distributed (Fig. S20); more TFs than would be expected by chance are in common for the regulation of intra-BTR genes, whilst the opposite is true for inter-BTR regulation. Thus, as long as different sets of TFs are activated or repressed, diverse BTRs will be coordinately transcribed or silenced. In addition, we studied the associations between BTRs and particular TFs. Fig. 6 shows a network of TFs (diamonds) linked with 31 specific BTRs (circles), in which each BTR is coloured according to the biological processes over-represented within it. For example, BTRs 209 and 258 are both associated with Hap3, Hap4 and Hap5 (Fig. 6, top left). These TFs, alongside Hap2, form the CCAAT-binding complex, which regulates respiratory functions^64–66^. Both BTRs are also associated with Sut1, which is involved in hypoxic gene expression^67^. BTR 209 is also associated with another regulator of gene expression in response to oxygen levels, Hap1^68^. As mentioned above, both BTRs are involved in respiration. Both BTRs also contain additional members; for instance, BTR 209 also contains Inh1, which inhibits ATP hydrolysis by the ATP synthase^69^. This is consistent with the regulation of complex IV and V of the oxidative phosphorylation pathway in order to maintain the proper balance on the proton gradient, and the complementarity of this relationship is shown visually in Fig. 7 for the two BTRs concerned. As another example, BTRs 318 and 381 are associated with Rpn4, which stimulates the expression of proteasome genes and hence influences protein degradation^70^. However, Rpn4 is also amongst the many TFs associated with cytoplasmic ribosome associated BTR 180, coupling both the synthesis and degradation of proteins; it activates the expression of the proteasome genes whereas it represses the ribosomal genes.

### Yeast builds stress-specific defenses by combining different BTRs

**Figure 8:**
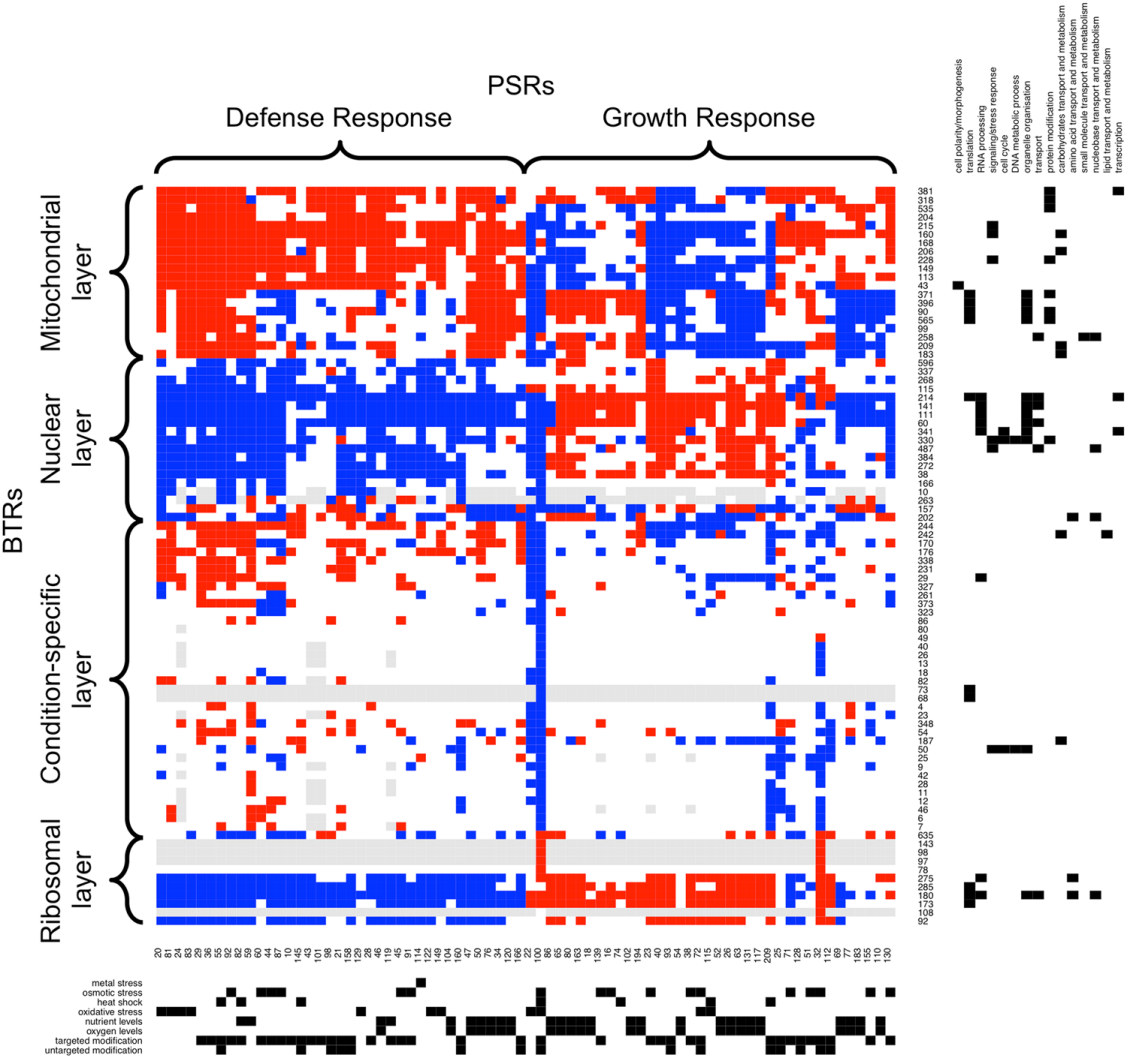
BTRs as stress-response functional blocks. Heatmap shows the changes of expression due to stress-response: activation (red), repression (blue), and no change (white). Each column is a PSR, whereas BTRs are shown in rows. Grey squares show when there was no data available. Outer black squares are used to describe the BTRs and PSRs in terms of Biological Processes involved in and stresses, respectively.

Next, we analysed relationships between BTRs and PSRs. Briefly, we calculated if BTR members were statistically over- or under-regulated in a particular PSR (see Methods for details). Subsequently, we clustered the results which revealed a high-level organization of BTRs into four coherent but different ‘layers’ (Fig. 8): three coordinated layers representing common mitochondrial, nuclear, and ribosomal responses, and a fourth condition-specific layer. The mitochondrial layer of response contains several BTRs that act on mitochondrial translation and carbohydrate metabolism. In addition, it also includes BTRs involved in protein modification: phosphorylation and proteolysis. The nuclear layer involved BTRs related to cell cycle progression, DNA transcription and RNA processing. The ribosomal layer of BTRs was involved in cytoplasmic translation. Intriguingly, this layer also included several BTRs containing Ty1 and Ty2 retrotransposons and delta-type LTRs. Some transposons are known to preferentially integrate near genes transcribed by RNA polymerase III^71,72^, such as tRNAs. Therefore, it is plausible that their expression increases when these Pol III-transcribed genes are highly expressed as a consequence of activated protein synthesis. These three layers of response contained BTRs that had significant expression differences in most PSRs. In contrast, the condition-specific layer contained BTRs with expression changes in relatively few PSRs. Their lack of functional homogeneity suggests they are necessary for stress-specific responses. It is noteworthy that the combined analysis reveals the existence of a mitochondrial ‘layer’, whereas we found no evidence of a global mitochondrial response in the analysis of PSRs. This implies that even if groups of mitochondrial genes respond coordinately, their changes of expression are rarely in the top or bottom of the differential gene lists and potentially they could be over-looked. In order to investigate this further, we tested if members of BTRs were in the subsets of RNAs experiencing the greatest expression fold changes. We considered a BTR as undergoing large significant change if more than half of its members were in the top/bottom 1%, 5% or 10% subsets identified during the PSR analysis. We only found 4 BTRs associated to the broad category of carbohydrate transport and metabolism (BTRs 160, 183, 206 and 209) that have great changes in more than one PSR (Fig. S21). Results presented in Fig. S21 demonstrate that this is a general trend; even if BTRs are consistently induced or repressed during the stress response (Fig. 8), in very few occasions do they contain many genes making the top or bottom of the differential expression list.

From the PSR perspective, the results point to two main global responses: one that involves the activation of mitochondria, and the repression of the nuclear and ribosomal BTR layers; and, a second showing opposite trends. Many of the experiments within the PSRs in this second global response involve shifts from anaerobic to aerobic growth, and also include yeast growing in chemostat cultures. The latter controls growth at a constant rate. We have therefore characterized these two global responses as corresponding to defense and growth respectively. The global responses did not split PSRs perfectly by the type of stress; however, it seems that many resource-limitation stresses (e.g. specific nutrient starvation) favoured a nuclear-ribosomal response, whereas oxidative stress and targeted yeast modifications (e.g. experimental setups designed to affect processes such as the organization of cell wall or chromatin, phosphorylation or ubiquitination state of proteins, signaling or transcription) triggered mainly mitochondrial responses.

### BTRs as a theoretical framework for the analysis of NGS data

Collectively, these results demonstrate that BTRs are functionally coherent gene groups whose expression changes in response to varying growth conditions can be rationalized in terms of deployment of these concerted blocks. This suggests that novel analyses of differential expression might be better analysed at the block-level instead of on a per-gene basis. As the use of microarrays has become less popular with the advent of NGS, we wished to validate the use of BTRs in the analysis of novel NGS data, generated in our own independent experiments^73^ (see Methods for details). Standard quality control tests confirmed that biological replicates clustered with their associated stress conditions, based on their gene expression and gene expression changes (Figs S22 and S23), and each experiment therefore constitutes an independent PSR for comparative purposes. Amino acid starvation produced the most dramatic changes in gene expression compared to the other stresses (FDR < 0.01): 1535 genes were up-regulated and 1427 mRNAs were down-regulated, with large expression fold changes in both directions for both high and low abundant transcripts (Figs S24 and S25, and S11 File). The comparatively modest effects of glucose starvation and oxidative stress may be due to the duration of the stresses and/or the concentration of peroxide used. The number of enriched or depleted transcripts within each stress experiment was similar; however, a functional enrichment analysis (Fisher test; FDR < 0.01) demonstrated that there were more GO Slim biological processes associated with depleted genes that there were with up-regulated genes (Fig. S26), particularly for the amino acid starvation experiment.

**Figure 9:**
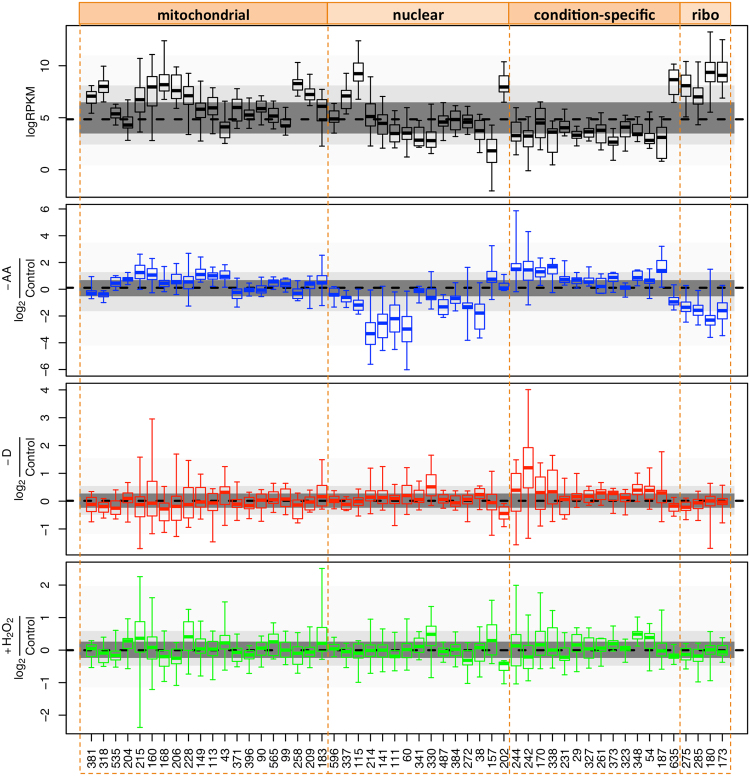
Transcript abundance and stress-response per BTR. Boxplots show maximum, minimum, interquartile range and median. Points-line shows the median value of all transcripts. Grey shading shows different inter-percentile ranges. BTRs are shown in the same order as in Fig. 8, grouped into the four major groupings from that figure. Some BTRs shown in Fig. 8 are missing because they did not contain enough members in the NGS data.

Direct comparison of equivalent microarray and NGS experiments is usually challenging because of technical and experimental reasons; e.g. the different range of expression detection, the different levels of noise associated with each methodology, and sometimes differences in statistical power due to insufficient number of biological replicates. Nevertheless, none of these reasons should invalidate the block-level approach. We therefore considered how BTRs might be used to rationalize our NGS-based stress response data using the most populated BTRs (≥10 RNAs). The changes in gene expression, measured by RNA-seq, with respect to normal growth conditions are shown in Fig. 9, displaying coordinated changes with respect to the BTRs. Additionally, there is also a clear pattern in the overall expression (Fig. 9 top panel); i.e. transcript abundance is clearly non-randomly distributed across the BTRs.

Considering the specific stresses, yeast responded to amino acid starvation with a global defense response; i.e. by shutting down the BTRs related to the nuclear and ribosomal responses, and expressing BTRs within the mitochondrial and condition-specific response layers. Amongst the activated condition-specific BTRs there were BTR 242 and BTR 187, which are involved in carbohydrate and lipid metabolism. Neither of the other stresses had a global effect on the nuclear, mitochondrial or ribosomal layers. Nevertheless, they both showed enrichment for transcripts within BTRs 330 and 348, and depletion for BTR 202, which contains transcripts involved in the biosynthesis of folate and purine nucleotides. Finally, glucose starvation also seemed to activate BTR 242, which is involved in lipids and monocarboxylic acid metabolisms. Taken together, our results imply that the coordinated stress-response is designed to ensure that genes within each BTR maintain a similar level of expression.

## Discussion

Based on analysis of gene expression changes on multiple yeast stress experiments since the early 2000s^10–20,23–25^, the general view is that stress responses include the induction of genes involved in defense against the specific stress applied^10,11^ and mitochondrial functions^10^, and also the shutdown of translation machinery^10,11^. Our meta-analysis has found that this general assumption is not always true and provides a framework for a more nuanced understanding (e.g. Figs 3 and 8). For instance, cells that are growing in a resources-limited environment show a growth response instead of the commonly-known defense response, even after being inflicted with osmotic or oxidative stress (e.g. PSR 72 and PSR 115). These cells were already growing at a low rate and can subsequently cope with a stress burden without further growth rate restriction. Also, as expected, the genomic background seems to be critical; e.g., PSR 38 shows how both KCl and glucose trigger a growth response in a yeast strain carrying a *HOG1* deletion.

When investigating the actual biological processes affected during the response to changing conditions, we found that stress-response genes are overexpressed in many environmental stresses as expected (Fig. 3). Nevertheless, ribosome biogenesis and cytoplasmic translation remain largely unaltered in many experiments. Furthermore, several PSRs (especially those associated with resource limitations) involved a decreased expression of multiple stress-response genes. Our independent RNA-seq analyses show a complementary picture; different stresses result in very different responses in terms of the biological processes affected (Fig. S26). Thus the decision whether to activate a defense response or continue on growing, as characterized in Fig. 8, probably depends on the growth conditions before the stimulus, the strength and type of the stimulus and the presence/absence of mutations. Nevertheless, it is worth noting that the final outcome not only depends on transcriptional changes, but also on various post-transcriptional events affecting the storage, stability and translation of RNAs^35,37,74–77^.

A further striking observation from our meta-analysis is one that will be familiar to experienced researchers in functional genomics; experimental protocol and/or the lab/human-factor can also have a greater effect on the transcriptional response than the actual stress (S1 and S5–S7 Files). For example, we have previously reported technical biases in target-identification within the context of protein-RNA interactions^36,78^ and quantitative proteomics^79^. Similar, biases have been reported for microarrays^39–41^ and RNA-seq^80^ which often report good Spearman (rank-based) correlations for inter-lab studies, but concede poorer results for individual transcripts and absolute measurements. As a consequence, greater overlap is expected from lists of up/down-regulated genes from experiments carried out under similar technical conditions (e.g. using identical chips), but smaller similarity is likely between completely unrelated experiments. Indeed, these observations motivated our strategy to consider overlapping, common gene sets derived from rank-ordered lists. This “consensus” approach has merits compared to those reporting genes identified by just a single group or technological platform. We believe that the concept of coordinately regulated functional blocks we introduce here may be useful for overcoming those technical biases and to act as a common frame of reference for highlighting the biological aspects of the response to stimuli (Fig. 8).

As noted throughout, many functional blocks comprise molecular machinery and enzymes common to certain metabolic pathways (Figs 4–9**)**, whose epistasis relationships underpin biological function^46–48,81^. As expected from previous module network analyses^43,44,63^, this interdependence can lead to correlated expression changes in response to changing conditions. However, members of BTRs also have similar transcript abundances (Fig. 9 top panel). This has implications for both systems and synthetic biology: altering the expression of one gene may trigger a regulatory response to restore the balance within the block, and should be considered in systems models or synthesis of novel pathways. Equally, the knowledge of co-ordinated blocks can be exploited to better estimate the biological variation between samples, and to establish the significance of differential expression (e.g. in an equivalent way as linkage-disequilibrium correlations may be used to estimate the effective number of tests in polymorphism association analyses)^82^.

Recent critical assessments of functional annotation algorithms point to superior abilities to predict molecular function above biological processes^83,84^. Dutkowski and coworkers have demonstrated improvements by integrating multiple data sets to infer biological processes^85^. Thus, the functional homogeneity of BTRs and their coherent regulation permits conjecture on the function of some uncharacterized proteins. For example BTR 263, which contains 13 poorly-characterised unnamed ORFs, is associated with two paralogous homeobox transcriptional repressors, Yhp1 and Yox1, that regulate genes expressed in M/G1 phase. Similarly, BTRs 60 and 330 are also associated with Yhp1 and Yox1. BTR 60 is mostly involved in ribosome organisation and RNA processing. BTR 330 is involved in DNA metabolic processes and cell cycle. Additionally, BTRs 263 and 330 are also associated with TFs that regulate transcription during G1/S transition: Mbp1, Swi6, and Swi4 (Fig. S27). This suggests a role in DNA/RNA metabolic processes during the cell cycle for the uncharacterized RNA members of BTR 263. Indeed, four of these RNAs resemble helicases.

Summing up, we believe that BTRs offer a new biologically relevant conceptual framework for the interpretation of gene expression analyses, which represent consensus blocks of co-ordinately regulated genes. Given that they represent distilled gene subsets, they are less prone to noise from single experiments, techniques or laboratories. They also permit rationalization of expression changes in the context of the stoichiometry requirements for protein complexes and metabolic pathways. We suggest that differential expression analyses should consider functional information such as that contained in BTRs to help understand, model and predict gene function and improve prediction of the biological processes that proteins are involved in.

## Electronic supplementary material

Supplementary information

Dataset 1

Dataset 2

Dataset 3

Dataset 4

Dataset 5

Dataset 6

Dataset 7

Dataset 8

Dataset 9

Dataset 10

Dataset 11
