# Supplementary material for: Archetypal transcriptional blocks underpin yeast gene regulation in response to changes in growth conditions
Source: Sci Rep. 2018 May 21;8:7949. doi: 10.1038/s41598-018-26170-5 (PMC5962585; doi:10.1038/s41598-018-26170-5)
Supplement: Supplementary file 1 — Supplementary information [file 41598_2018_26170_MOESM1_ESM.pdf]

SUPPLEMENTARY INFORMATION

Archetypal transcriptional blocks underpin yeast gene regulation in response to changes in growth conditions

David Talavera[1*], Christopher J Kershaw[2], Joseph L Costello[2,#a], Lydia M Castelli[2,#b], William Rowe[3,#c], Paul FG Sims[4], Mark P Ashe[2], Chris M Grant[2], Graham D Pavitt[2*], Simon J Hubbard[3*].

* Corresponding authors

david.talavera@manchester.ac.uk (DT)

graham.pavitt@manchester.ac.uk (GDP)

simon.hubbard@manchester.ac.uk (SJH)

Supplementary Figures in this file

S1	Decision tree in the assignment of instances to new or existing clusters.	4
S2	Relationship between clusters if changing the correlation threshold.	6
S3	Relationship between clusters if changing the linkage method.	7
S4	Relationship between clusters if changing the linkage method.	8
S5	Relationship between clusters if changing the linkage method.	9
S6	Relationship between clusters if applying normalization techniques to the downloaded data.	10
S7	Relationship between clusters if applying normalization techniques to the downloaded data.	11
S8	Analysis of common transcription regulation within and between BTRs.	12
S9	Characterization of PSRs.	13
S10	Response consistency within PSRs (top/bottom 10% RNAs).	14
S11	Response consistency within PSRs (top/ bottom 5% RNAs).	15
S12	Response consistency within PSRs (top/ bottom 1% RNAs).	16
S13	Biological processes affected by consistent stress response (top/ bottom 10% RNAs).	17
S14	Biological processes affected by consistent stress response (top/ bottom 1% RNAs).	19
S15	Characterization of BTRs.	21
S16	Biological processes overrepresented within BTRs (whole heatmap).	22
S17	Biological processes overrepresented within BTRs (zoomed in).	24
S18	Functional similarity within BTRs.	26
S19	Significance of interactions within BTRs.	27
S20	Regulation of transcription within and between BTRs.	28
S21	BTRs suffering extreme expression changes.	29
S22	Multi-dimensional scaling of transcriptomes from yeast grown under different stress conditions.	30
S23	Similarity of transcriptional response to stress (stress vs. control).	31
S24	Common stress responses.	32
S25	Magnitude of transcriptional stress-response.	33
S26	Biological processes affected by stress response.	34
S27	Use of BTRs for functional annotation.	35

Other files containing supporting information

S1 File. General description of data and clustering results.

S2 File. Matrix containing the log-fold changes for each experiment.

S3 File. Matrix containing the Pearson correlations between experiments.

S4 File. Matrix containing the Pearson correlations between RNAs.

S5 File. Alternative PSR clusters.

S6 File. In-detail description of PSRs analysed in the paper.

S7 File. Composition of PSR subsets analysed in the paper.

S8 File. Biological processes associated with subsets of PSRs.

S9 File. Composition of BTRs analysed in the paper.

S10 File. Biological processes associated with BTRs.

S11 File. edgeR results for NGS-based transcriptomic analyses.

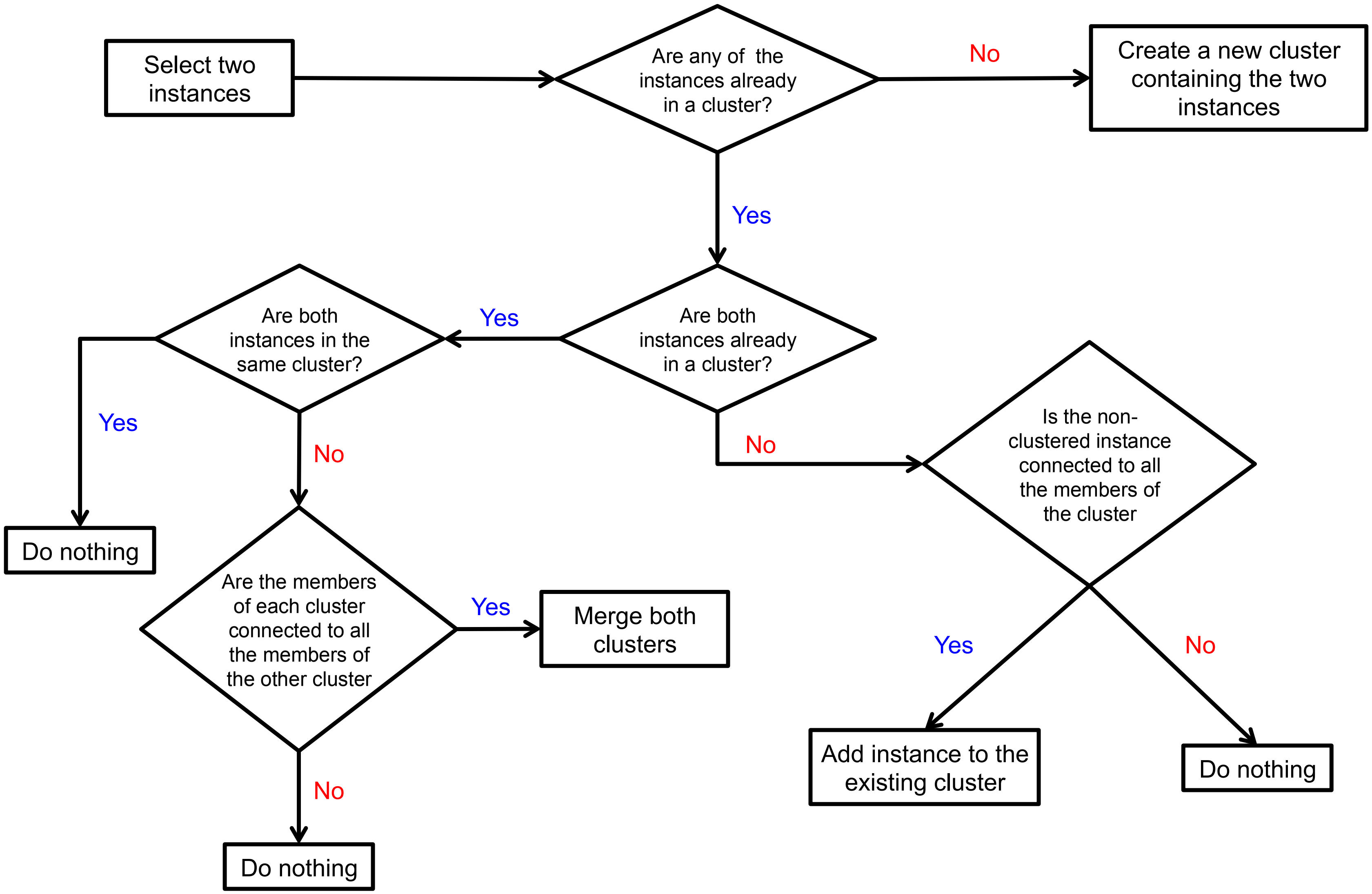
Select two instances
Are any of the instances already in a cluster?
No
Create a new cluster containing the two instances
Yes
Are both instances already in a cluster?
Yes
Are both instances in the same cluster?
Yes
Do nothing
No
Are the members of each cluster connected to all the members of the other cluster
Yes
Merge both clusters
No
Do nothing
No
Is the non-clustered instance connected to all the members of the cluster
Yes
Add instance to the existing cluster
No
Do nothing

S1 Fig. Decision tree in the assignment of instances to new or existing clusters.

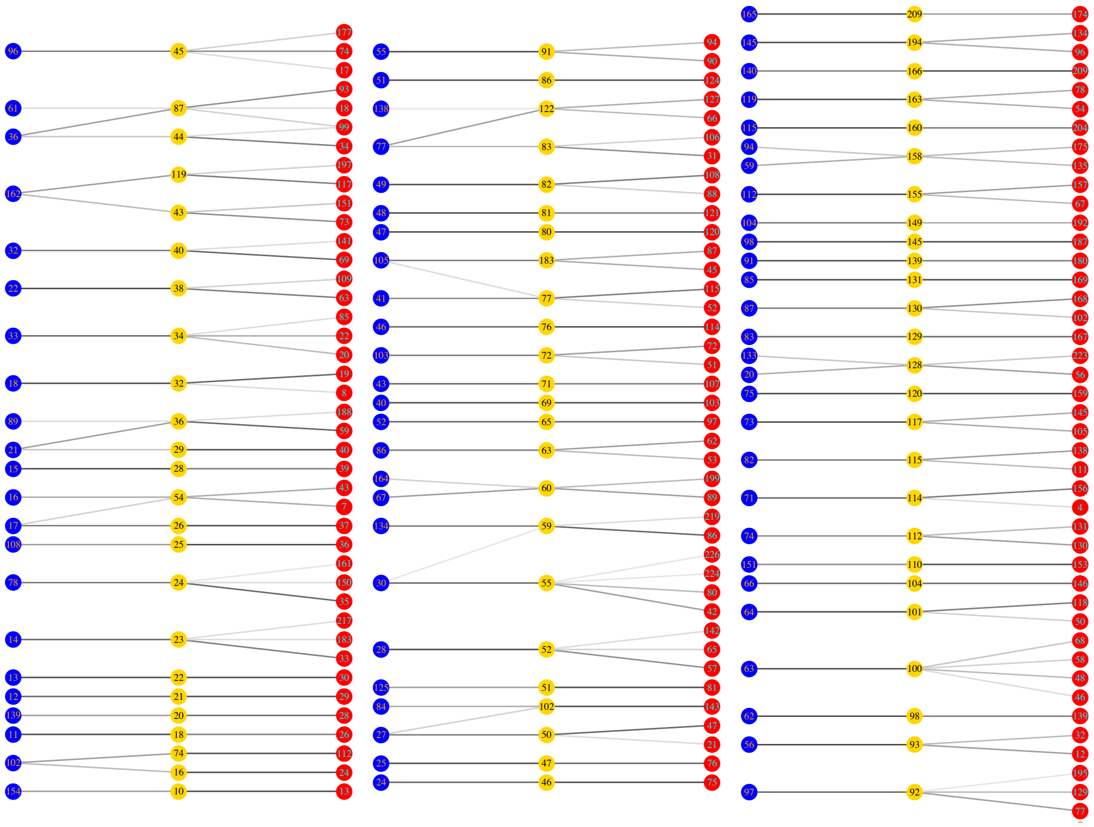

S2 Fig. Relationship between clusters if changing the correlation threshold. 0.4, in blue; 0.5, in yellow; 0.6 in red. The darkness of the lines represent the similarity of the clusters (Jaccard Index).

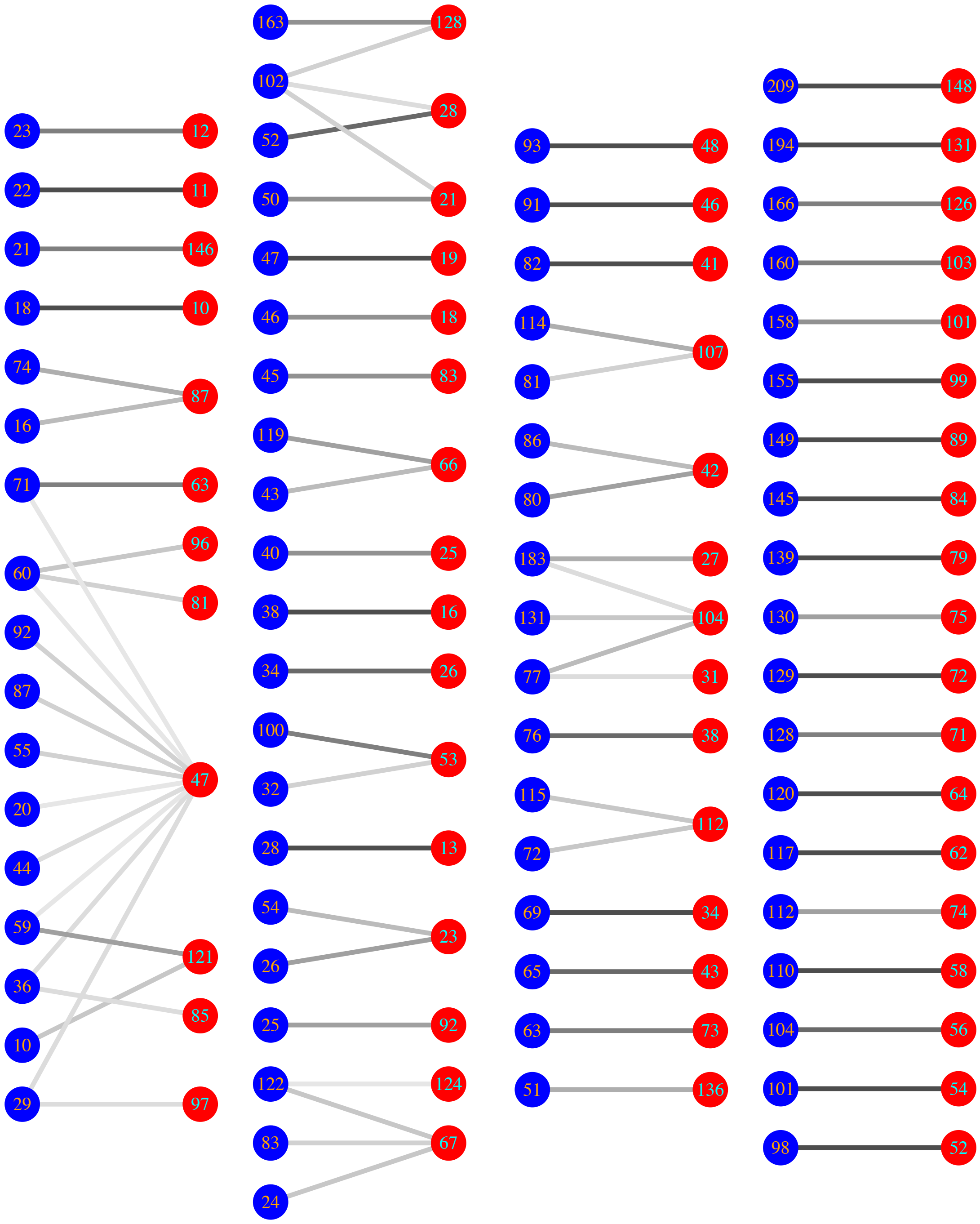

S3 Fig. Relationship between clusters if changing the linkage method. In red, the method described in the main text. In blue, clusters generated when requiring that new members do not decrease the mean of correlations beyond a particular threshold: 0.5 in this example. The darkness of the lines represent the similarity of the clusters (Jaccard Index).

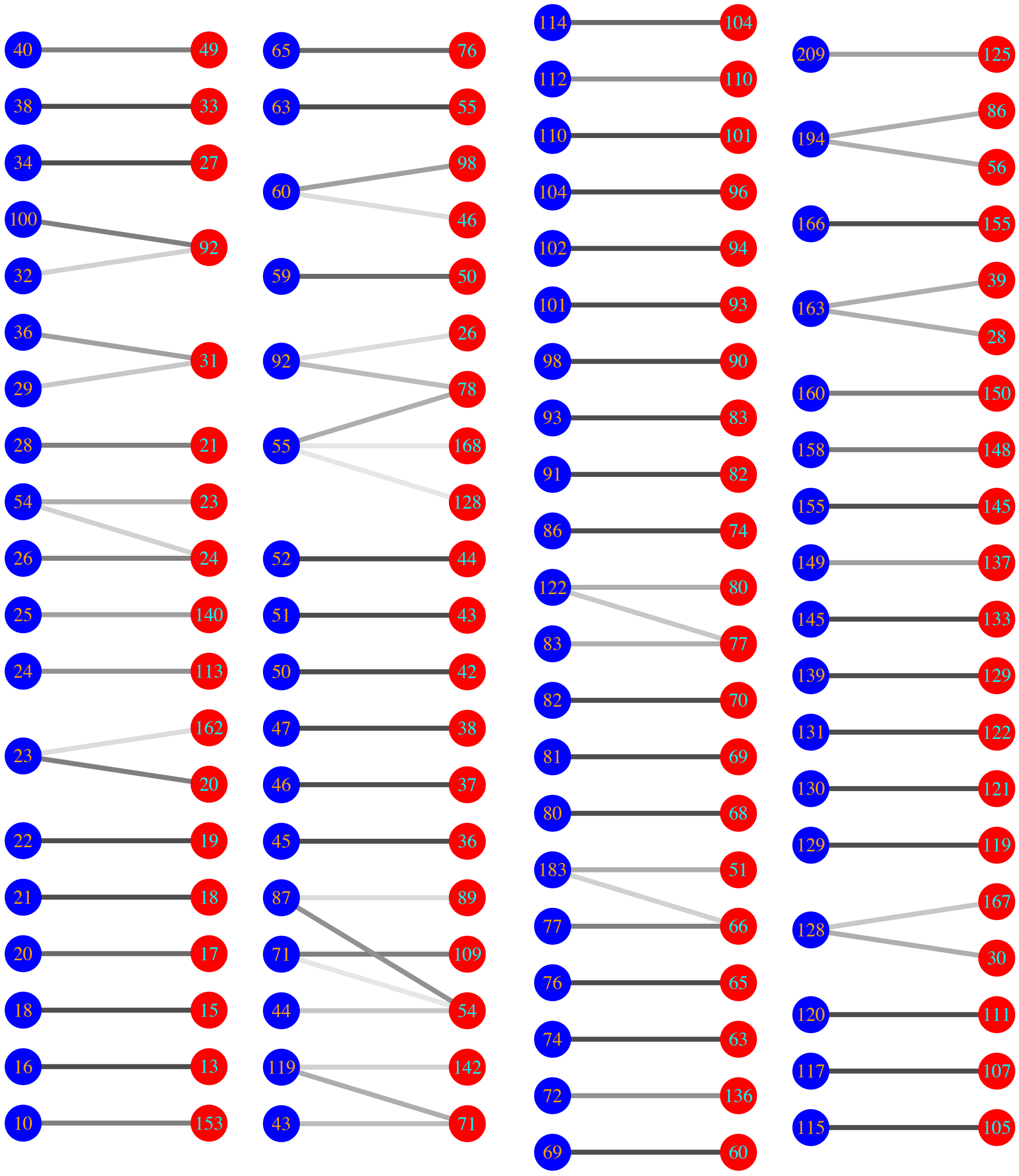

S4 Fig. Relationship between clusters if changing the linkage method. In red, the method described in the main text. In blue, clusters generated when requiring that new members do not decrease the mean of correlations beyond a particular threshold: 0.6 in this example. The darkness of the lines represent the similarity of the clusters (Jaccard Index).

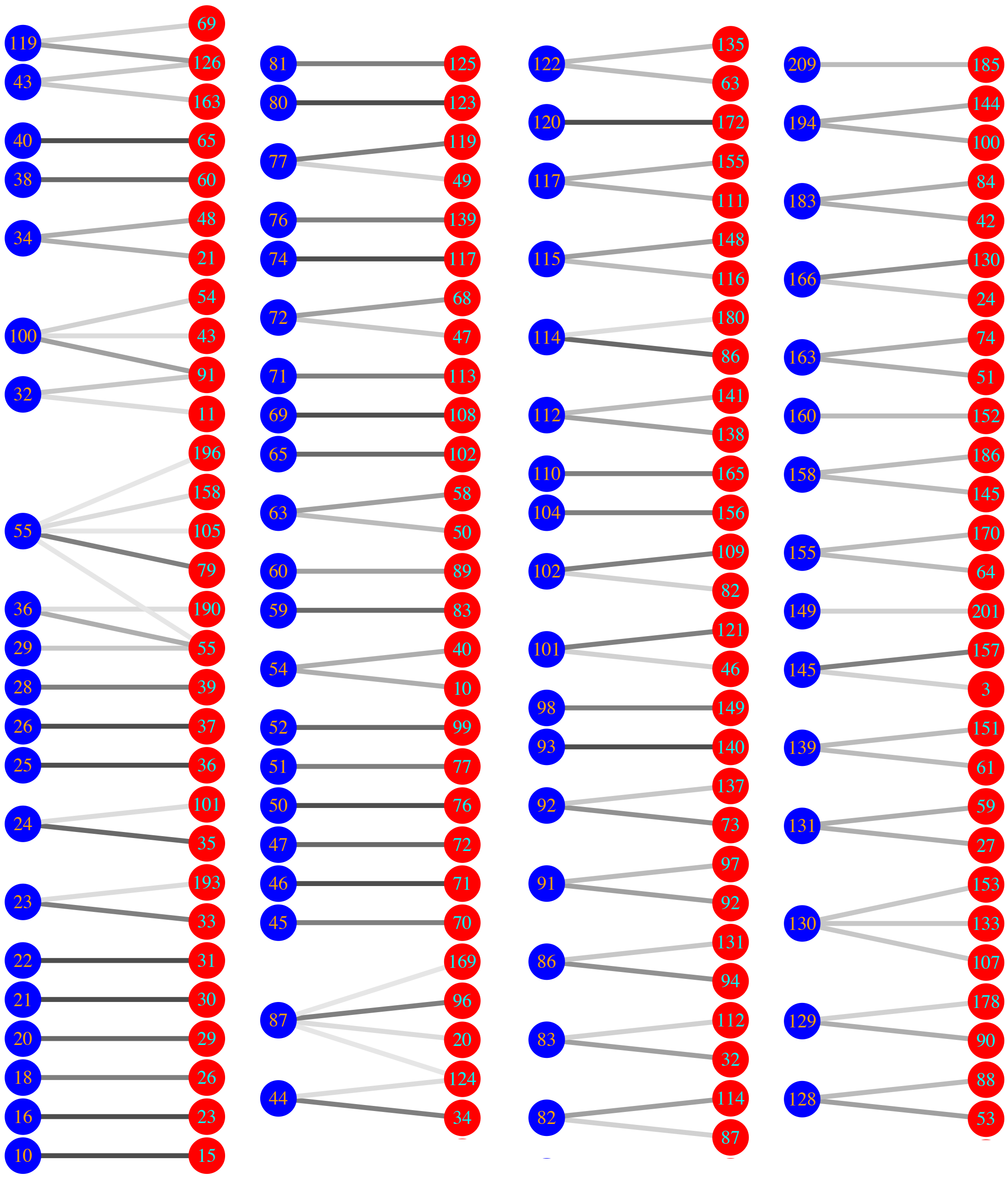

S5 Fig. Relationship between clusters if changing the linkage method. In red, the method described in the main text. In blue, clusters generated when requiring that new members do not decrease the mean of correlations beyond a particular threshold: 0.7 in this example. The darkness of the lines represent the similarity of the clusters (Jaccard Index).

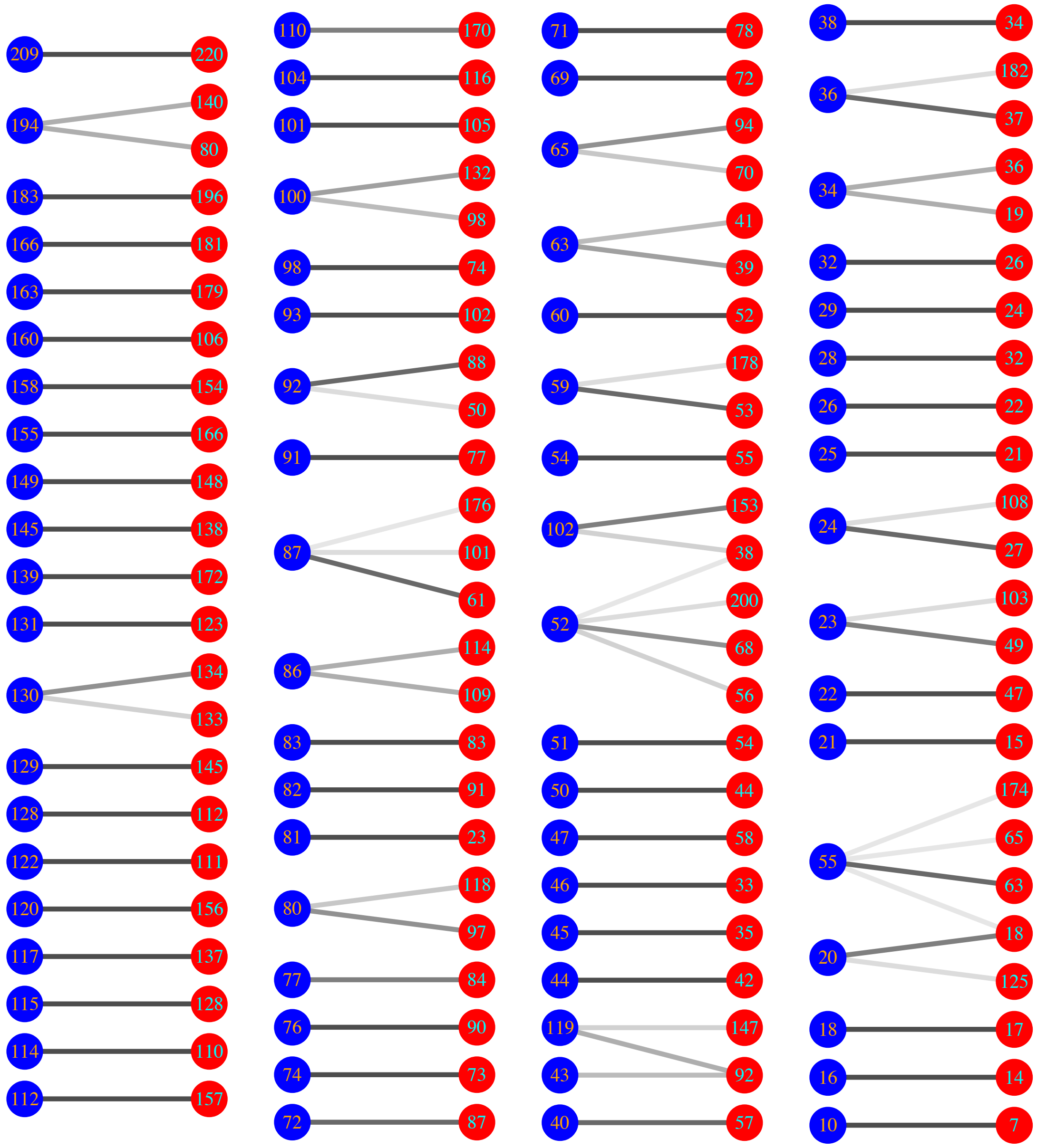

S6 Fig. Relationship between clusters if applying normalization techniques to the downloaded data. In red, the method described in the main text. In blue, clusters generated normalizing data with a Rank-based quantile normalization approach prior to the correlation calculation. The darkness of the lines show how similar are the clusters (Jaccard Index).

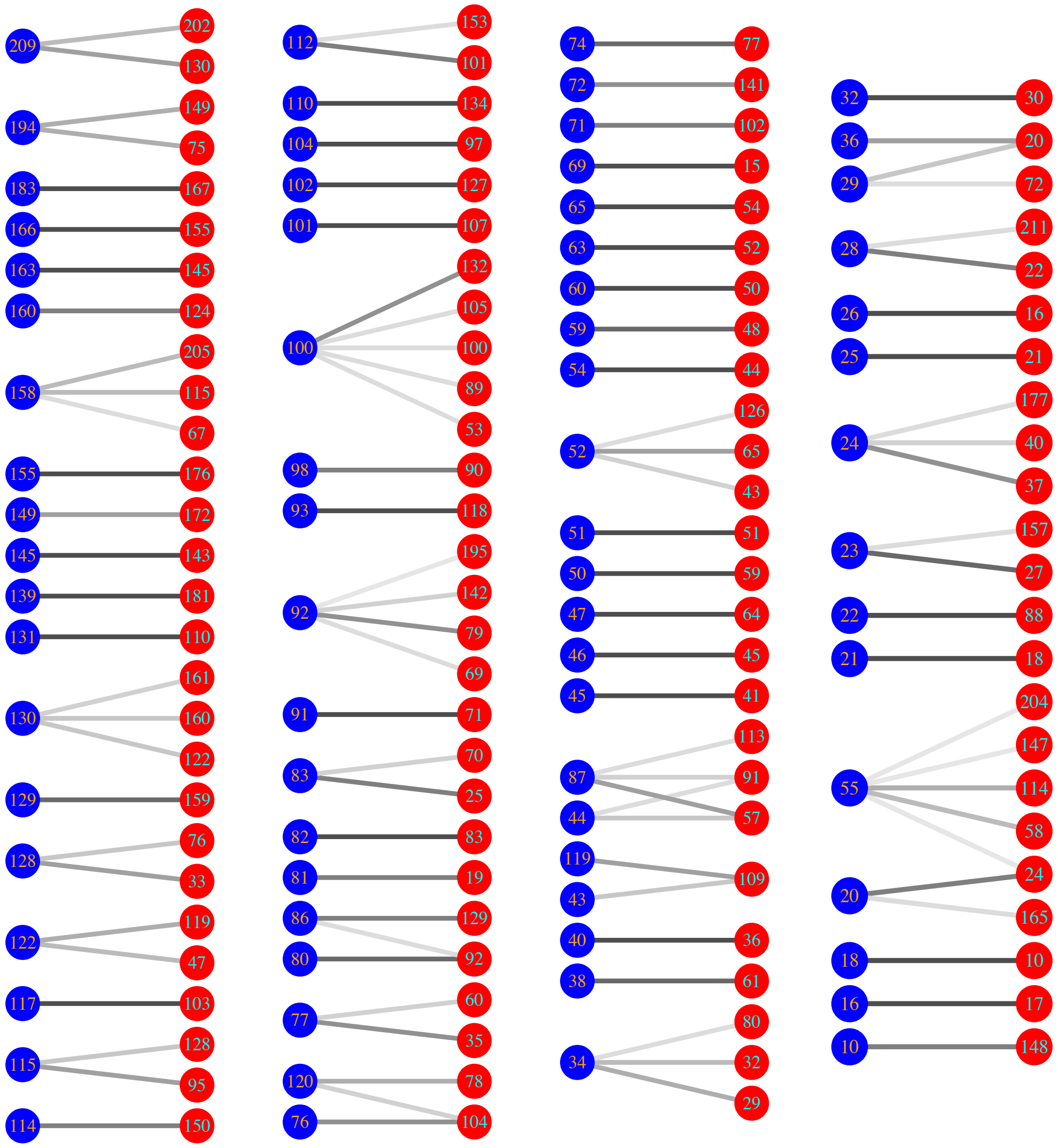

S7 Fig. Relationship between clusters if applying normalization techniques to the downloaded data. In red, the method described in the main text. In blue, clusters generated normalizing data with a Spline-based quantile normalization approach prior to the correlation calculation. The darkness of the lines show how similar are the clusters (Jaccard Index).

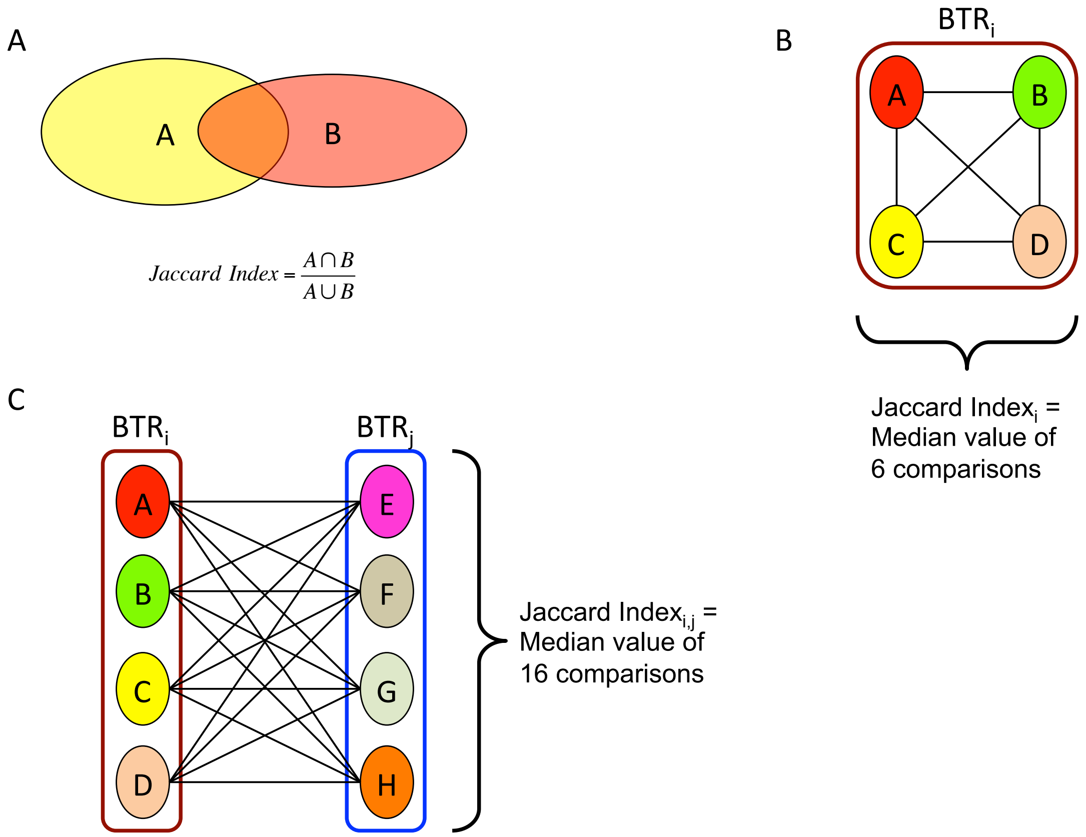

S8 Fig. Analysis of common transcription regulation within and between BTRs. A. Diagram showing the calculation of the Jaccard Index between two sets. B. Analysis of common TFs within a BTR containing 4 RNAs: the black lines represent the pairwise comparisons between the different sets of TFs (one for each RNA). C. Analysis of common TFs between two BTRs: the black lines represent all the pairwise comparisons between sets in different BTRs.

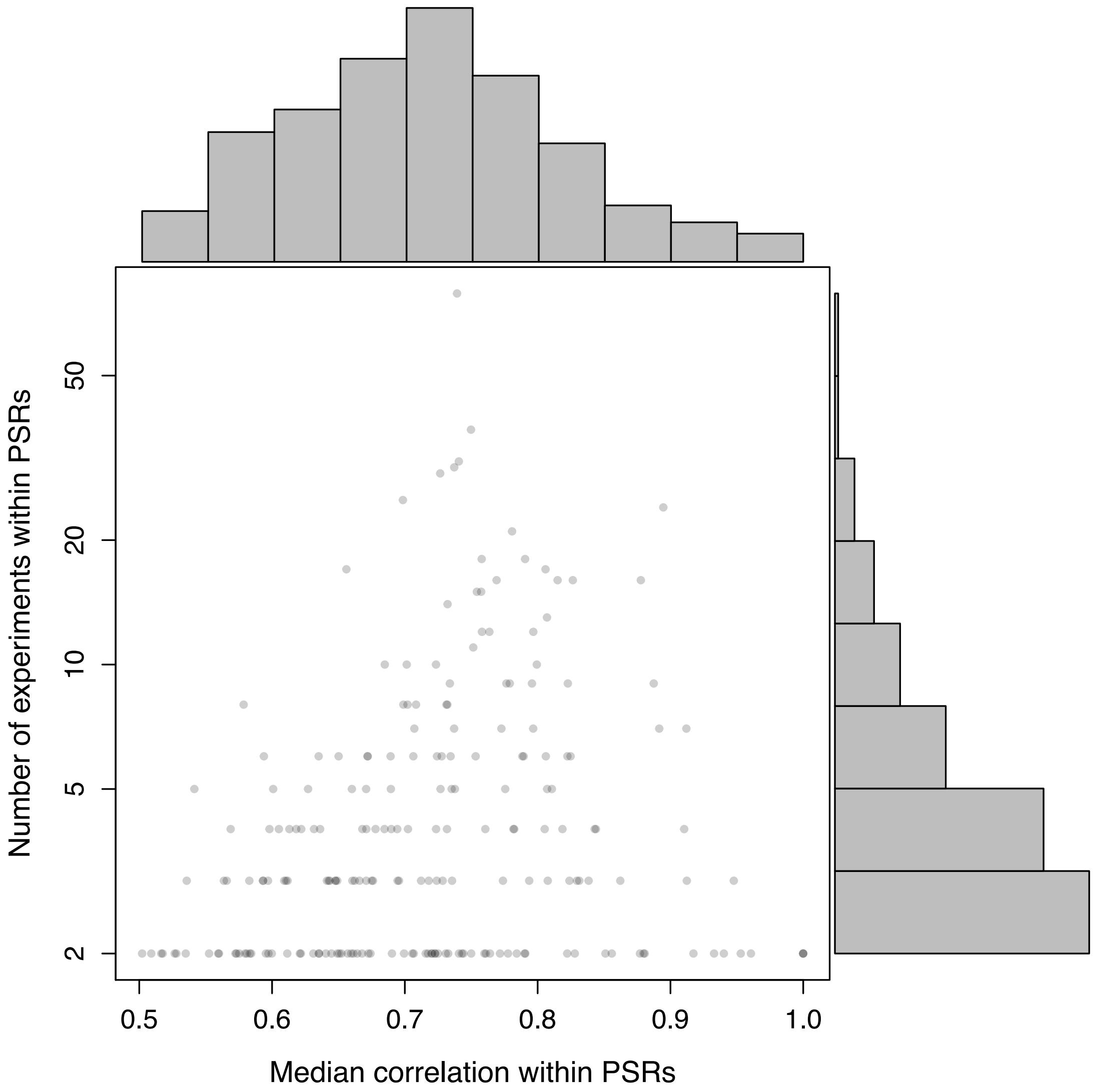

S9 Fig. Characterization of PSRs. Scatterplot shows the distribution of PSRs based on their within-group similarity (median of pairwise correlations within a PSR), and number of experiments within. Intensity of grey dots represents the number of overlapping points: the darker, the greater the number. Top and side histograms show the overall distribution of PSRs according to each variable.

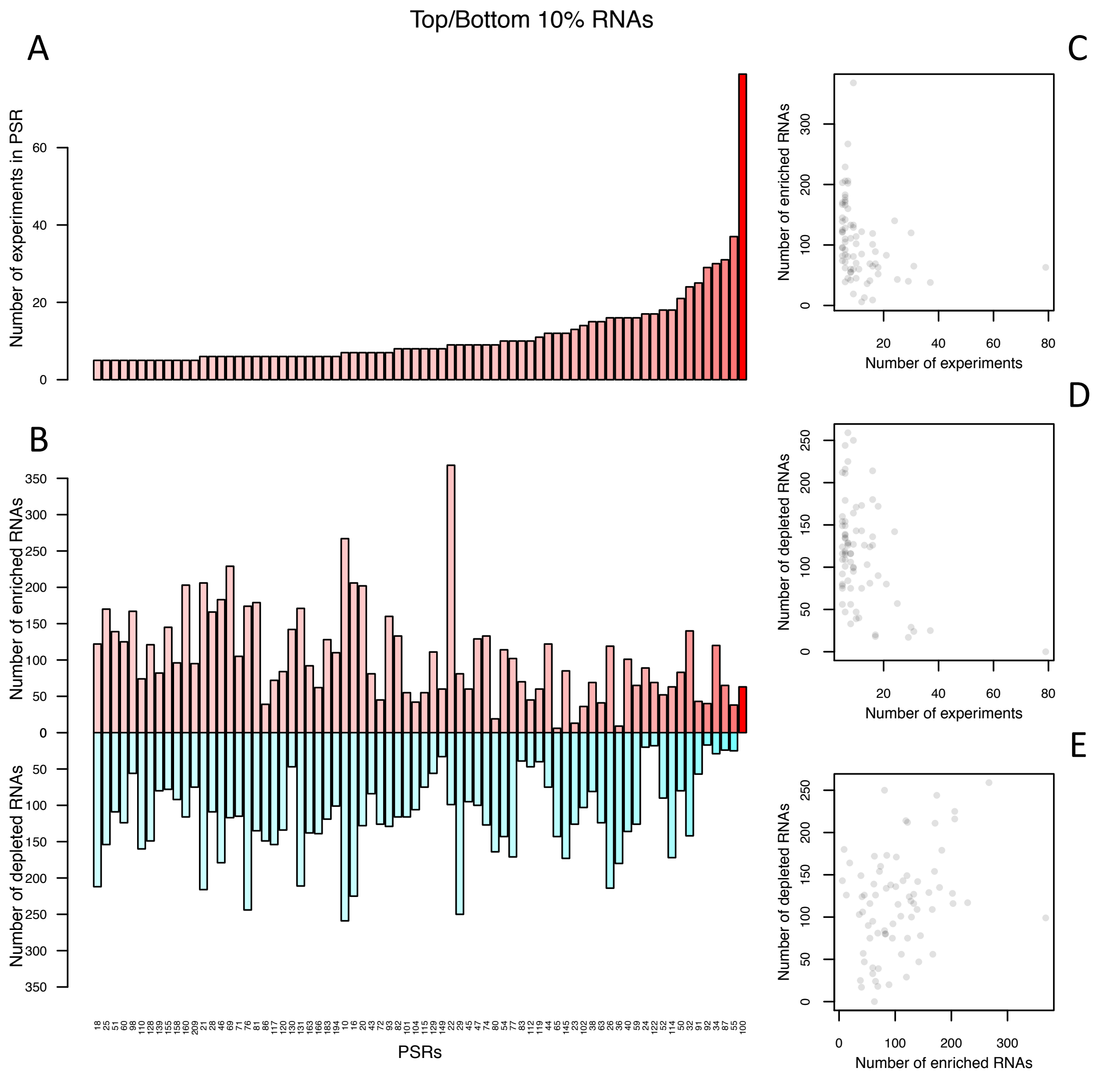

S10 Fig. Response consistency within PSRs (top/bottom 10% RNAs). A. Number of experiments within each PSR, sorted in increasing order. B. Number of consistently depleted and enriched RNAs within each PSR, ordered as in A. C-E. Relationship between number of experiments within PSR, number of enriched transcripts within PSR and number of depleted transcripts within PSR.

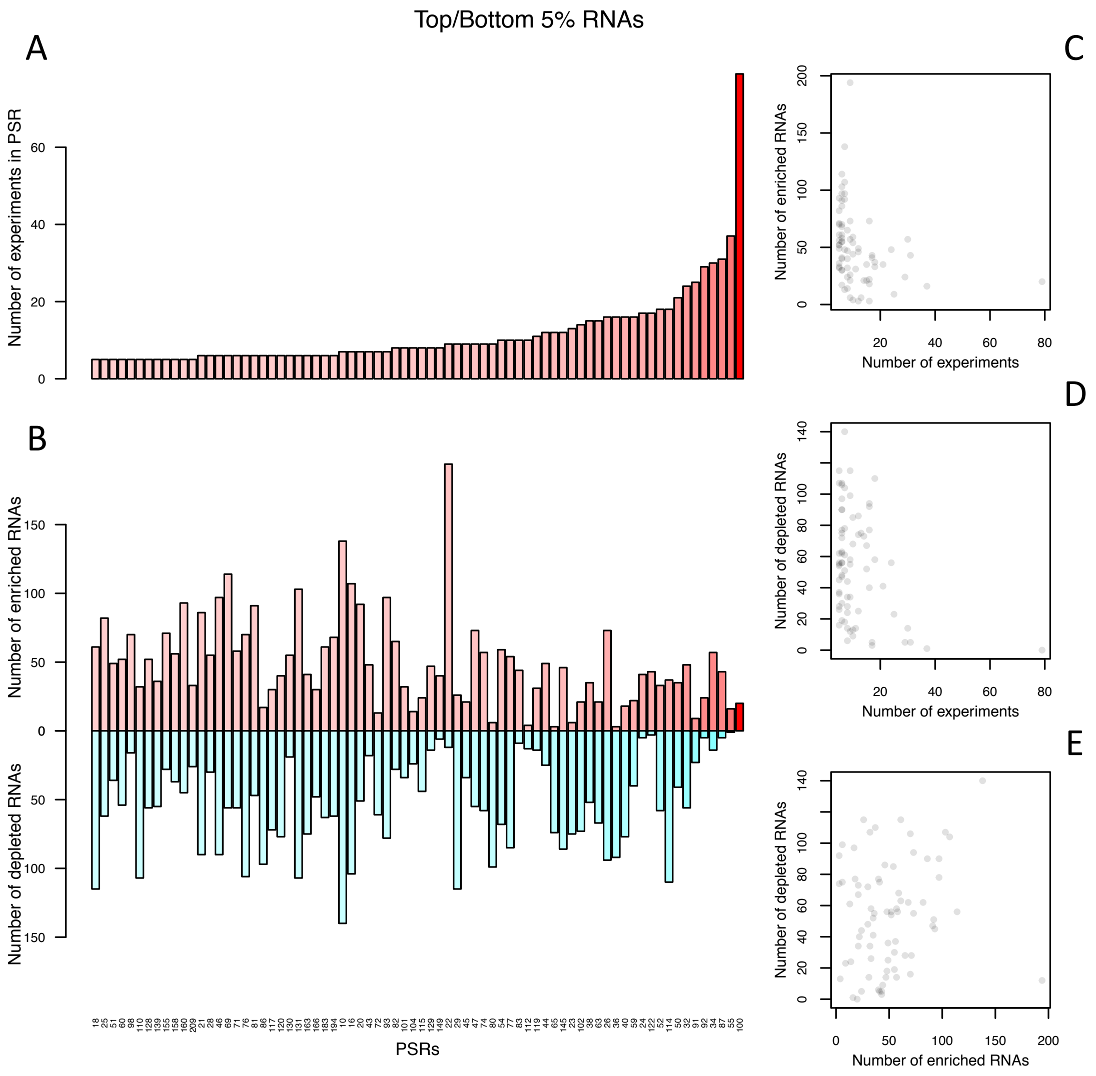

S11 Fig. Response consistency within PSRs (top/ bottom 5% RNAs). A. Number of experiments within each PSR, sorted in increasing order. B. Number of consistently depleted and enriched RNAs within each PSR, ordered as in A. C-E. Relationship between number of experiments within PSR, number of enriched transcripts within PSR and number of depleted transcripts within PSR.

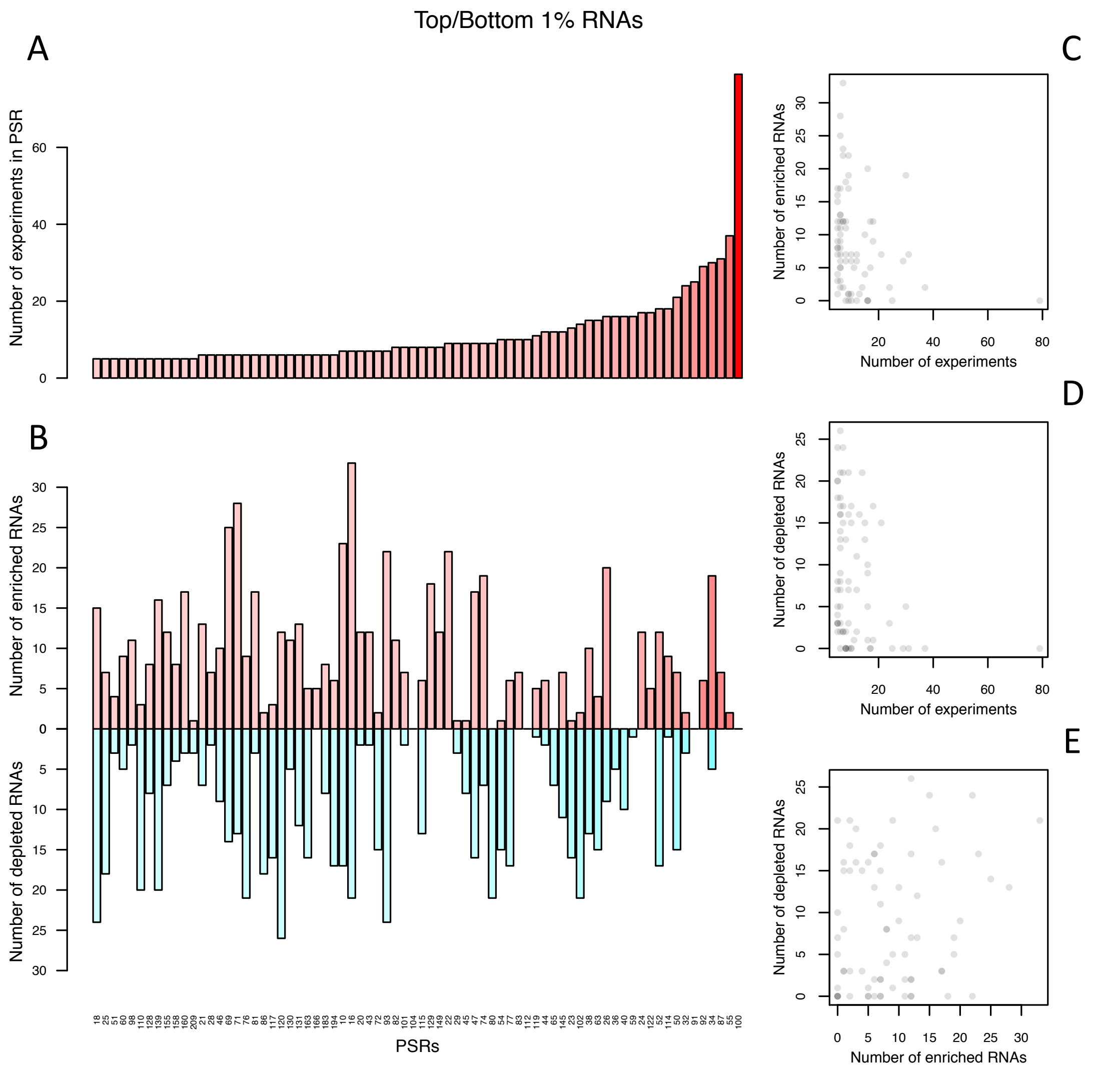

S12 Fig. Response consistency within PSRs (top/ bottom 1% RNAs). A. Number of experiments within each PSR, sorted in increasing order. B. Number of consistently depleted and enriched RNAs within each PSR, ordered as in A. C-E. Relationship between number of experiments within PSR, number of enriched transcripts within PSR and number of depleted transcripts within PSR.

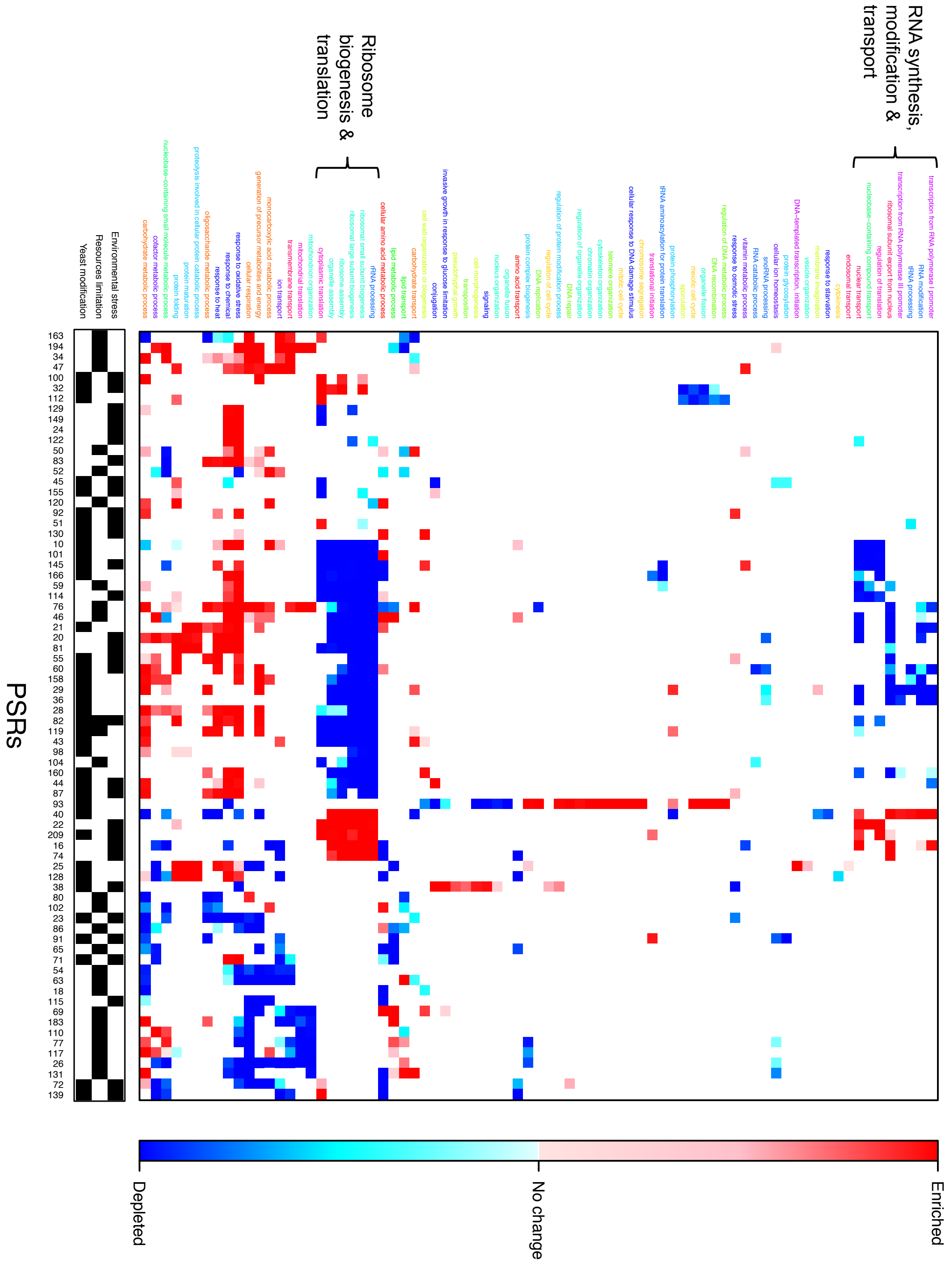
RNA synthesis, modification & transport
Ribosome biogenesis & translation
Environmental stress
Resources limitation
Yeast modification
PSRs
Depleted
No change
Enriched

S13 Fig. Biological processes affected by consistent stress response (top/ bottom 10% RNAs). GO Slim terms overrepresented in the set of 10% most-enriched transcripts (red) or the set of 10% most-depleted transcripts (blue). All colouring as in **Fig 3**. The black squares describe the type of stresses the PSRs suffered. The order of both PSRs and biological processes depends on the hierarchical clustering of Euclidean distances calculated along the columns and rows respectively.

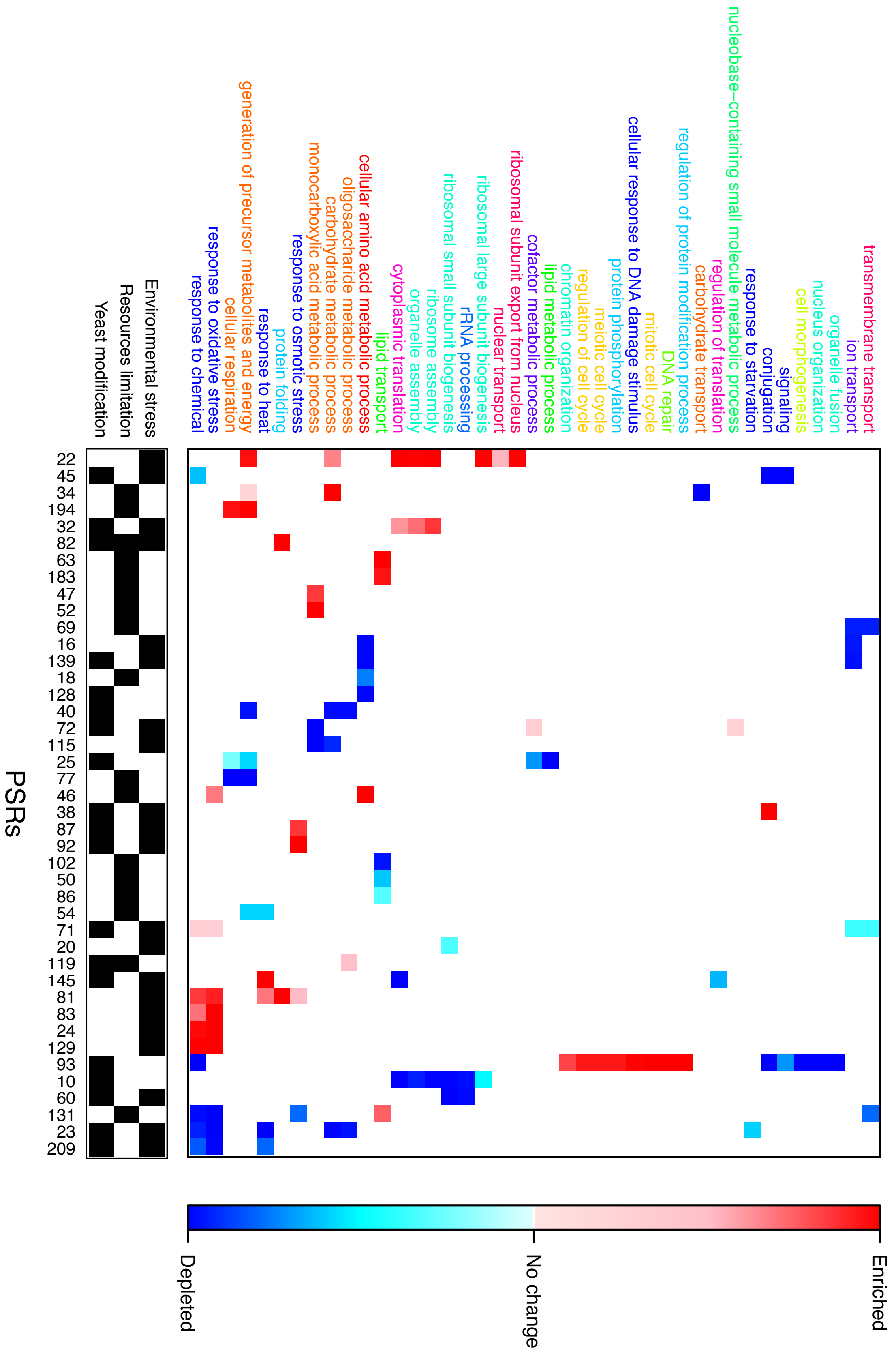

Environmental stress
Resources limitation
Yeast modification
PSRs
response to chemical
response to oxidative stress
cellular respiration
generation of precursor metabolites and energy
response to heat
protein folding
response to osmotic stress
monocarboxylic acid metabolic process
carbohydrate metabolic process
oligosaccharide metabolic process
cellular amino acid metabolic process
lipid transport
cytoplasmic translation
organelle assembly
ribosome assembly
ribosomal small subunit biogenesis
rRNA processing
ribosomal large subunit biogenesis
nuclear transport
ribosomal subunit export from nucleus
cofactor metabolic process
lipid metabolic process
chromatin organization
regulation of cell cycle
meiotic cell cycle
mitotic cell cycle
protein phosphorylation
cellular response to DNA damage stimulus
DNA repair
regulation of protein modification process
carbohydrate transport
regulation of translation
nucleobase−containing small molecule metabolic process
response to starvation
conjugation
signaling
cell morphogenesis
nucleus organization
organelle fusion
ion transport
transmembrane transport
22
45
34
194
32
82
63
183
47
52
69
16
139
18
128
40
72
115
25
77
46
38
87
92
102
50
86
54
71
20
119
145
81
83
24
129
93
10
60
131
23
209
Depleted
No change
Enriched

S14 Fig. Biological processes affected by consistent stress response (top/ bottom 1% RNAs). GO Slim terms overrepresented in the set of 1% most-enriched transcripts (red) or the set of 1% most-depleted transcripts (blue). All colouring as in **Fig 3**. The black squares describe the type of stresses the PSRs suffered. The order of both PSRs and biological processes depends on the hierarchical clustering of Euclidean distances calculated along the columns and rows respectively.

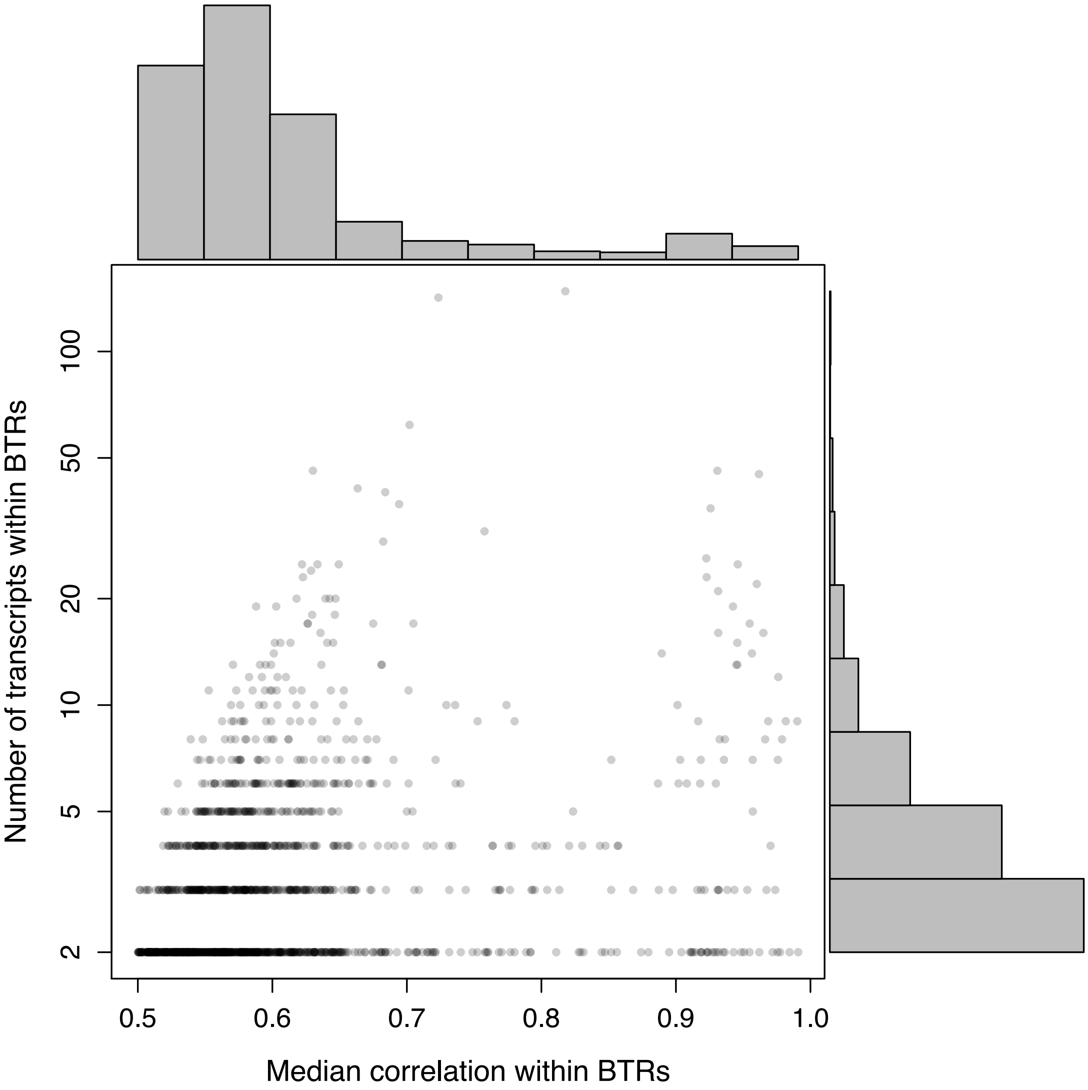

S15 Fig. Characterization of BTRs. Scatterplot shows the distribution of BTRs based on their within-group similarity (median of pairwise correlations within a BTR), and number of transcripts within. Intensity of grey dots represents the number of overlapping points: the darker, the greater the number. Top and side histograms show the overall distribution of BTRs according to each variable.

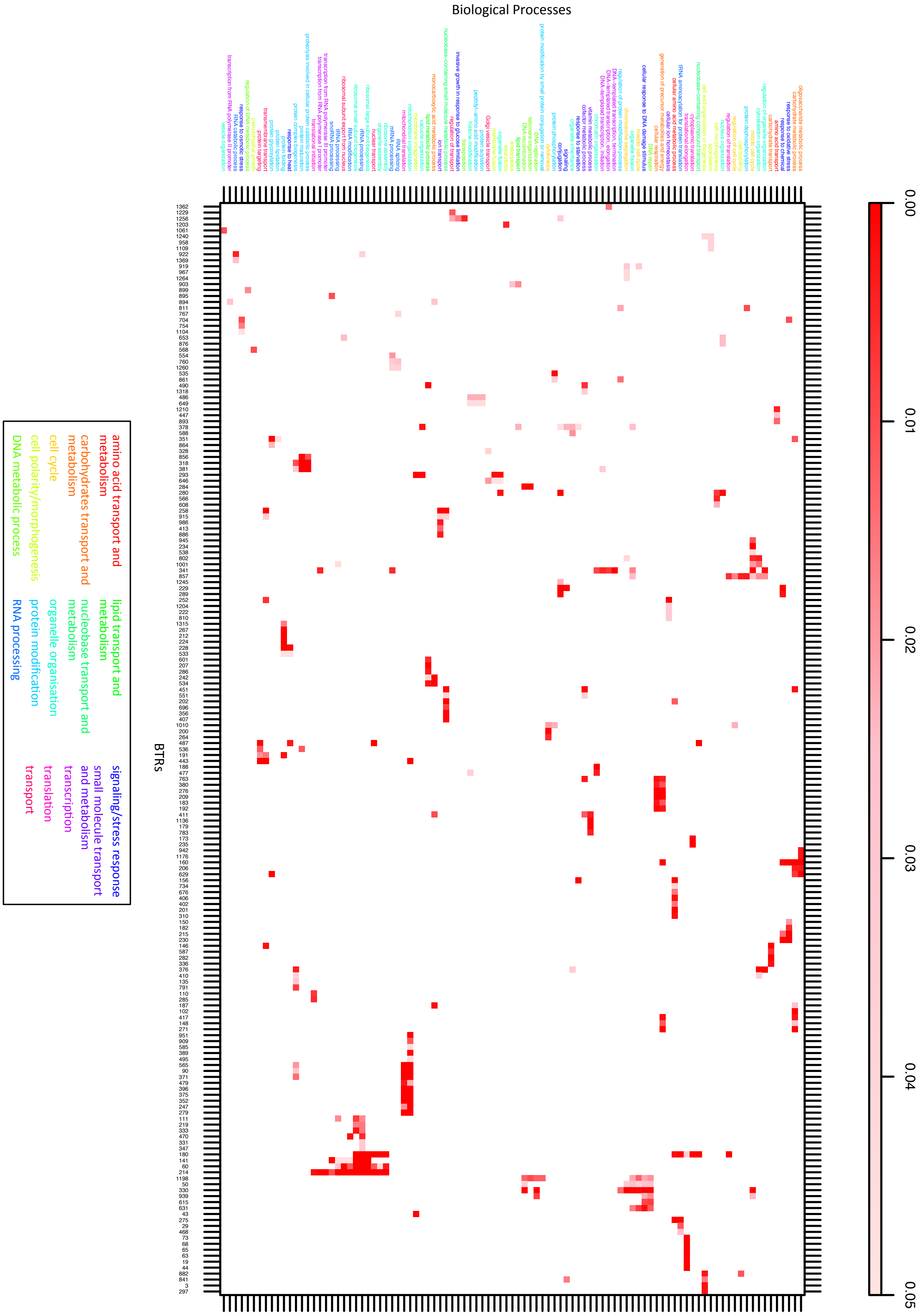
Biological Processes
BTRs
amino acid transport and metabolism
carbohydrates transport and metabolism
cell cycle
cell polarity/morphogenesis
DNA metabolic process
lipid transport and metabolism
nucleobase transport and metabolism
organelle organisation
protein modification
RNA processing
signaling/stress response
small molecule transport and metabolism
transcription
translation
transport
0.00
0.01
0.02
0.03
0.04
0.05

S16 Fig. Biological processes overrepresented within BTRs (whole heatmap). Red intensity represents the FDR value. Terms are grouped in 15 categories, and their name coloured accordingly. Zoomed in version of some areas of the heatmap in **S13 Fig**.

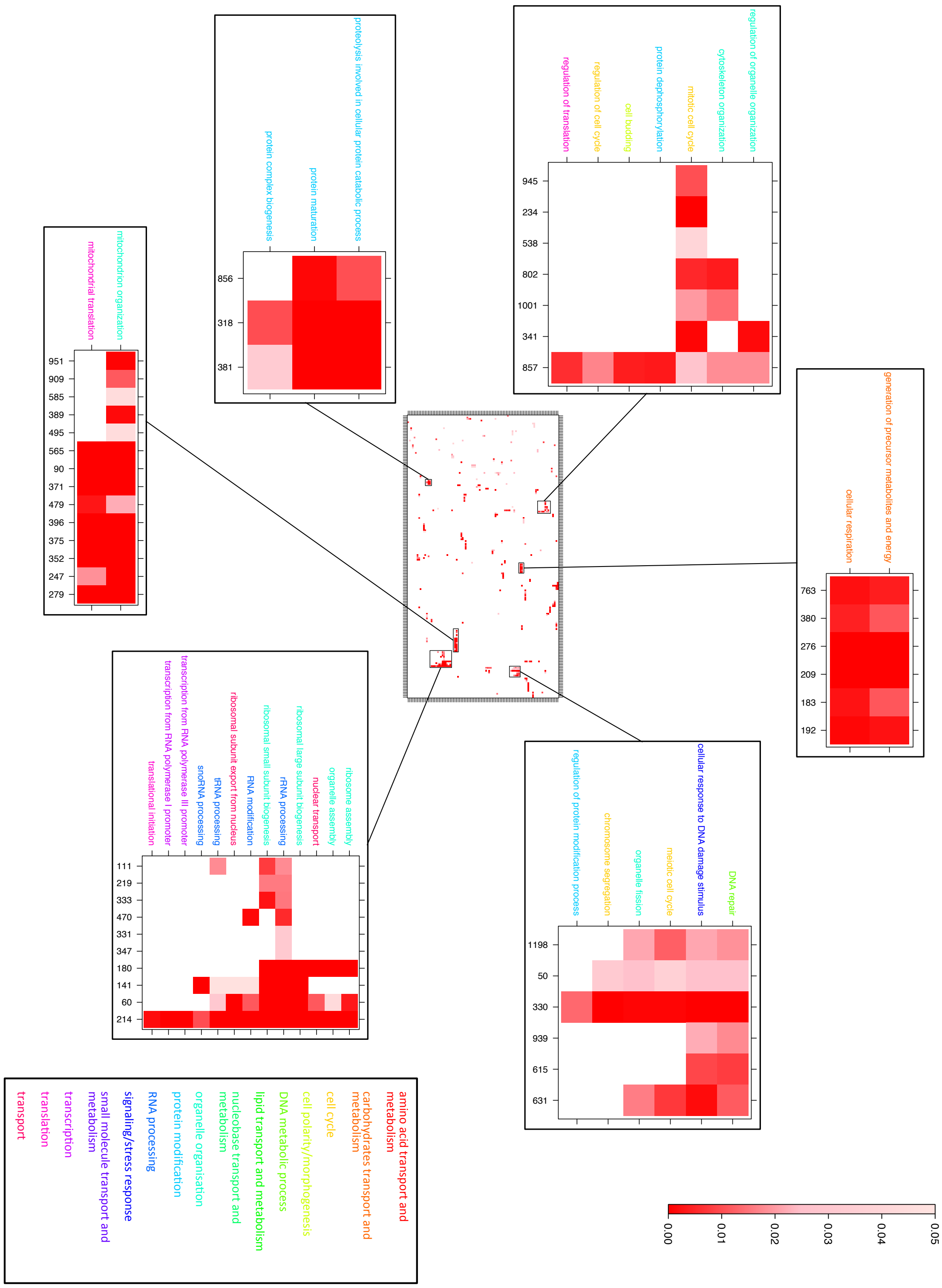
mitochondrial translation
mitochondrion organization
protein complex biogenesis
protein maturation
proteolysis involved in cellular protein catabolic process
regulation of translation
regulation of cell cycle
cell budding
protein dephosphorylation
mitotic cell cycle
cytoskeleton organization
regulation of organelle organization
cellular respiration
generation of precursor metabolites and energy
translational initiation
transcription from RNA polymerase I promoter
transcription from RNA polymerase III promoter
snoRNA processing
tRNA processing
ribosomal subunit export from nucleus
RNA modification
ribosomal small subunit biogenesis
rRNA processing
ribosomal large subunit biogenesis
nuclear transport
organelle assembly
ribosome assembly
regulation of protein modification process
chromosome segregation
organelle fission
meiotic cell cycle
cellular response to DNA damage stimulus
DNA repair
amino acid transport and metabolism
carbohydrates transport and metabolism
cell cycle
cell polarity/morphogenesis
DNA metabolic process
lipid transport and metabolism
nucleobase transport and metabolism
organelle organisation
protein modification
RNA processing
signaling/stress response
small molecule transport and metabolism
transcription
translation
transport
0.00
0.01
0.02
0.03
0.04
0.05

S17 Fig. Biological processes overrepresented within BTRs (zoomed in). Details of BTRs participating in similar biological processes. Red intensity represents the FDR value. Terms are grouped in 15 categories, and their name coloured accordingly.

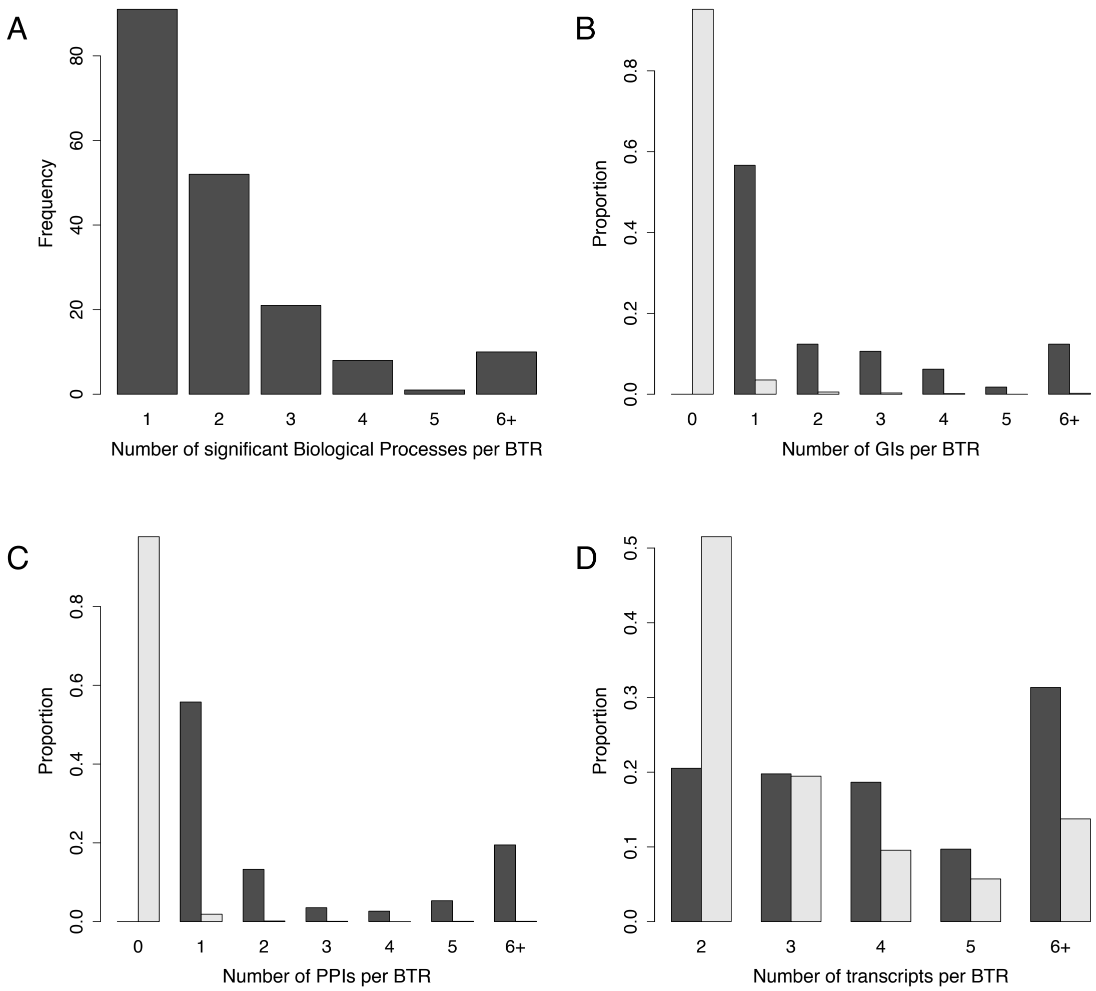

S18 Fig. Functional similarity within BTRs. A Number of GO Slim Biological Processes that are significantly enriched within each BTR. B. Number of significant (dark grey) and non- significant (light grey) genetic interactions within BTR. C. Number of significant (dark grey) and non-significant (light grey) protein-protein interactions within BTR. D. Number of transcripts per BTR for BTRs having a significant enrichment in A-C (dark grey) or not having any enrichment in A-C (light grey).

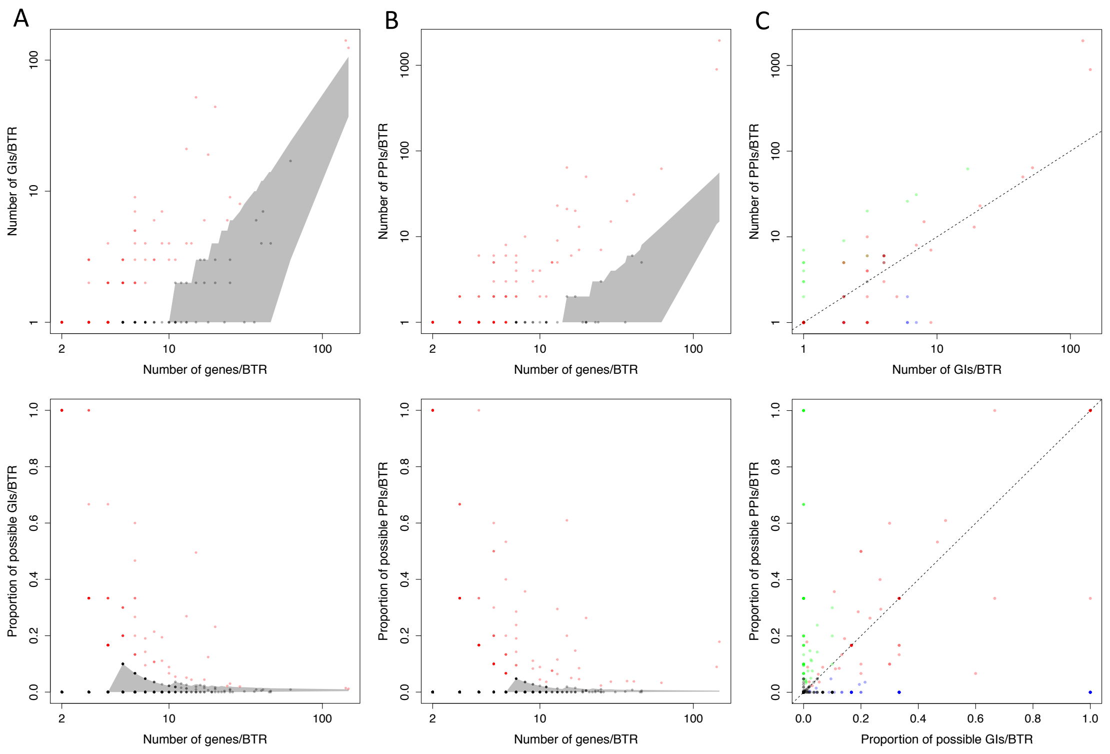

S19 Fig. Significance of interactions within BTRs. Number of (and proportion of all possible) genetic (A) and physical (B) interactions per BTR compared to the size of BTR. Significant cases are shown in red. Random expectation was calculated through a resampling process, and is shown as a grey shade (90% confidence intervals for the expectation). C. Number of (and proportion of all possible) physical and genetic interactions within BTRs. BTRs may have a significant overrepresentation of physical (green), genetic (blue) or both interactions (red). BTRs with no overrepresentation are shown in grey. Significance was established as p-value < 0.05. The intensity of the colour is proportional to the number of cases represented by a single point.

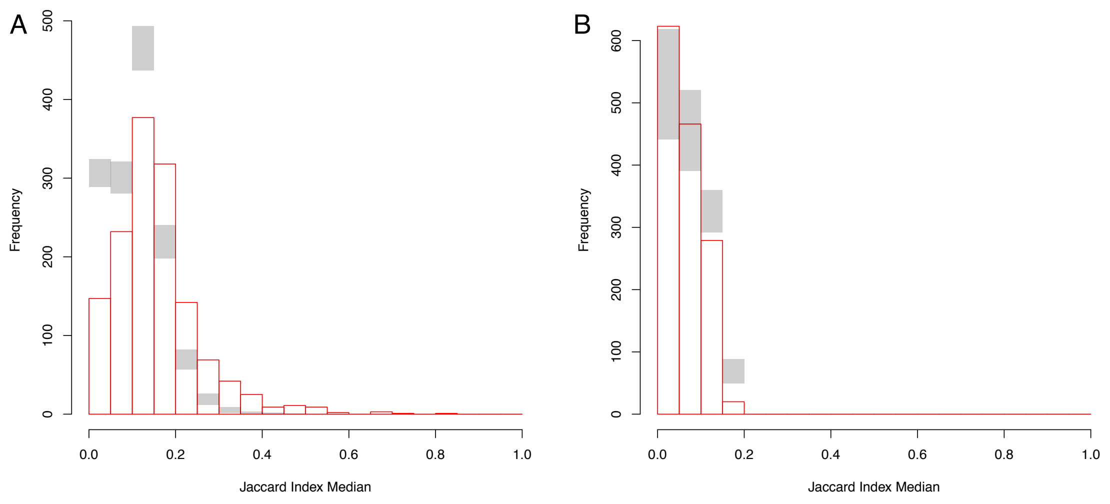

S20 Fig. Regulation of transcription within and between BTRs. Histograms show the distribution of overlapping sets of transcription factors involved in regulating the expression of transcripts measured as the median of Jaccard Indices (number of TFs controlling the expression of both genes/number of TFs controlling the expression of either of the genes). The grey shades show the expected tip of the histogram bars calculated using resampling. A. Within BTRs. B. Between BTRs.

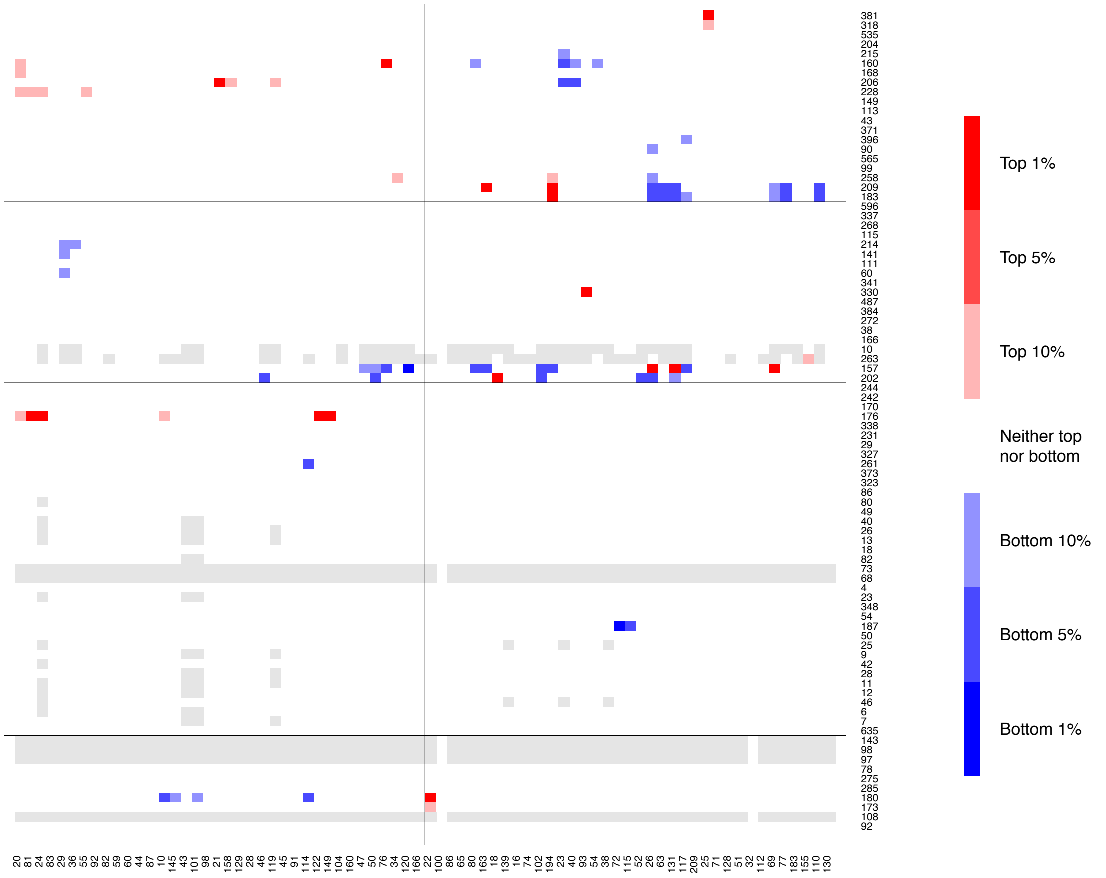

S21 Fig. BTRs suffering extreme expression changes. Heatmap shows if more than half of the members of a specific BTR are in one of the subsets of great changes of a particular PSR. Red shows membership of the subsets of induced RNAs, whereas blue shows membership of the subsets of repressed RNAs. The brighter the colour, the more extreme the percentile. PSRs and BTRs are ordered as they are in **Fig 8**: PSRs in columns, BTRs in rows. Grey squares show when there was no data available. Black lines divide the heatmap in the different global responses and response layers shown in **Fig 8**.

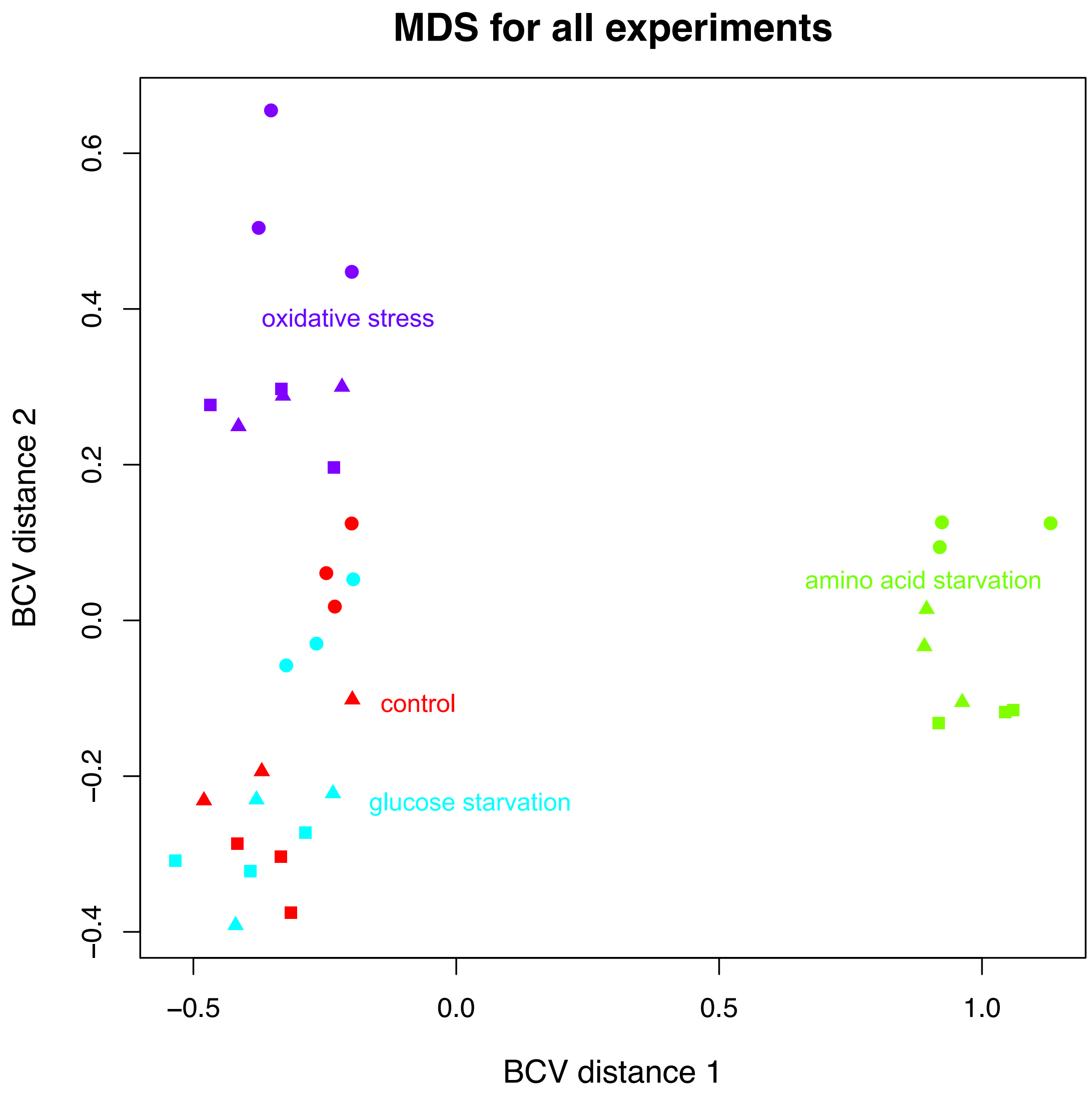

S22 Fig. Multi-dimensional scaling of transcriptomes from yeast grown under different stress conditions. Control (red); amino acid starvation (green); glucose starvation (blue); and, oxidative stress (purple). The point shapes represent the tags used in the experiment: eIF4E (squares); eIF4G1 (triangles); and, eIF4G2 (circles). It demonstrates that transcriptomes cluster because of stress instead of tag.

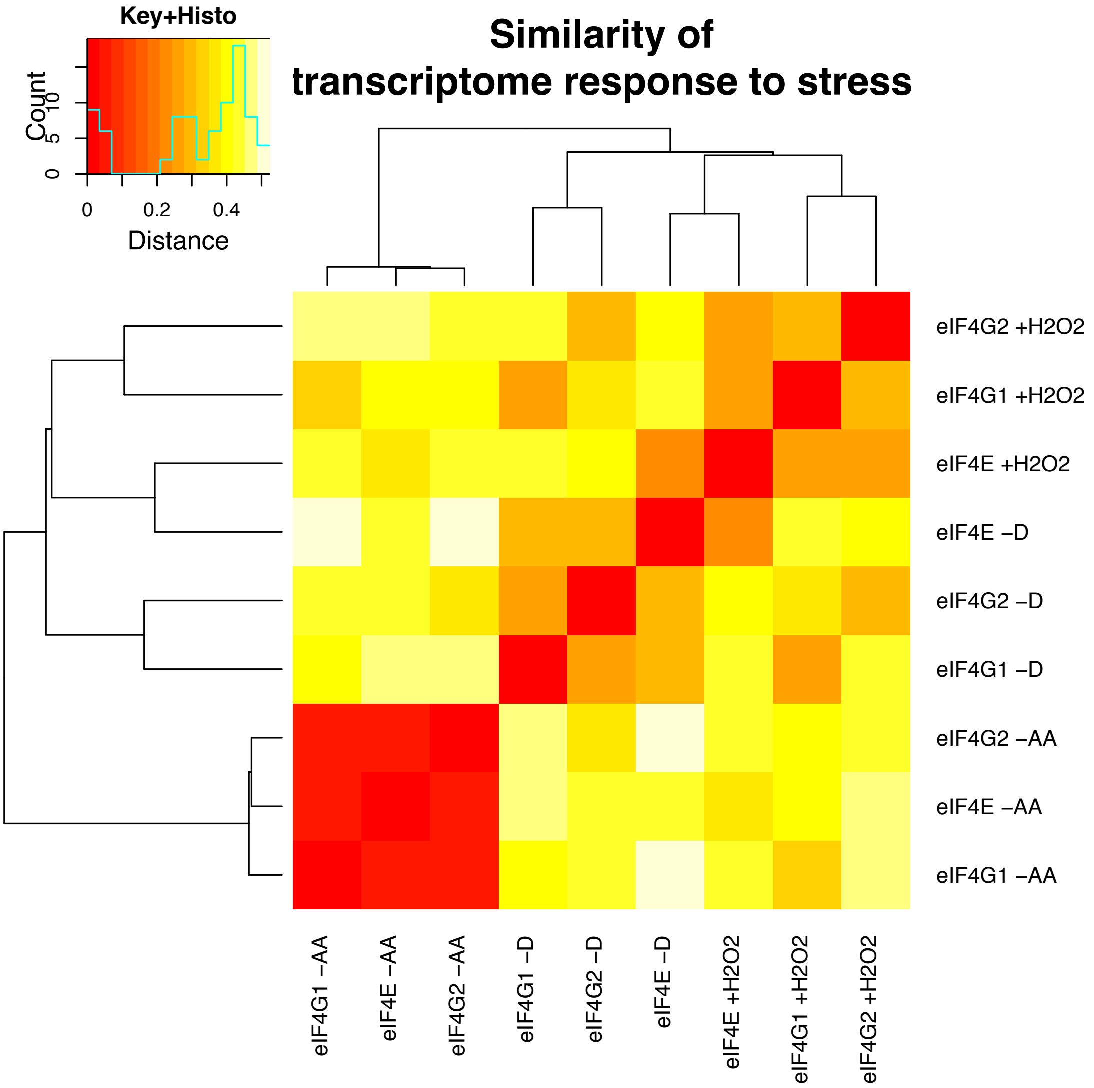

S23 Fig. Similarity of transcriptional response to stress (stress vs. control).

It demonstrates that stress responses cluster because of stress instead of tag.

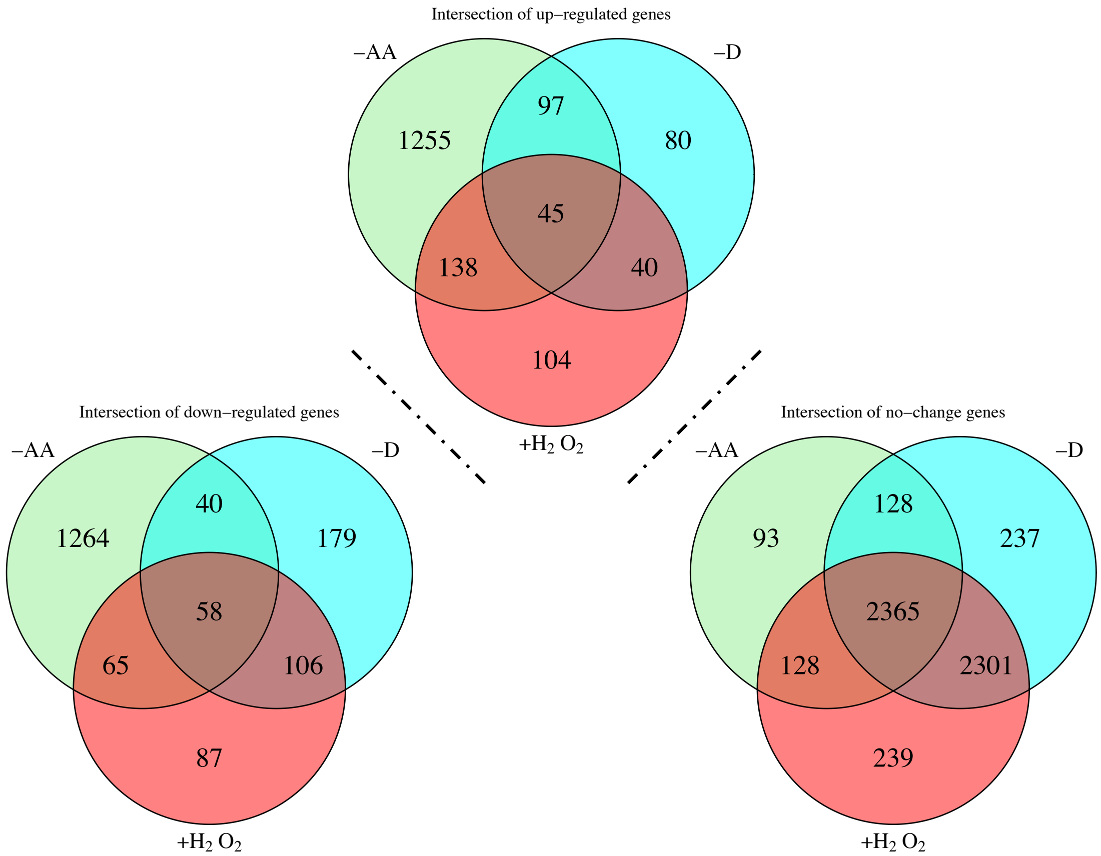

S24 Fig. Common stress responses. Overlapping sets of up-regulated genes (A), down- regulated genes (B), and genes with no statistical change (C) following 3 different stresses: amino acid starvation, glucose starvation, and oxidative stress. Statistical significance set at FDR 1%.

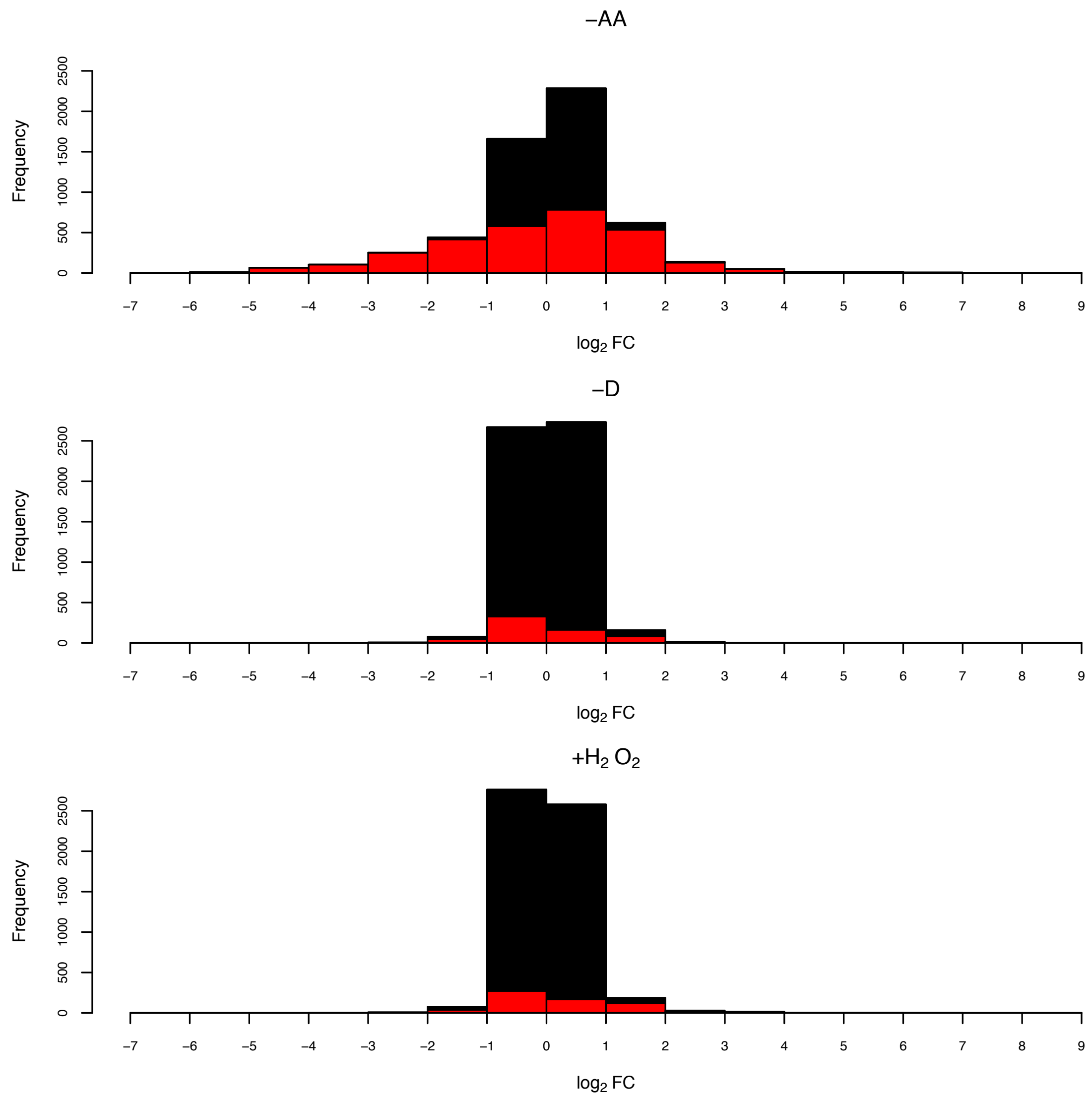

S25 Fig. Magnitude of transcriptional stress-response. Histograms show the distribution of log_2 fold-changes. Significant differentially-expressed transcripts are shown in red, the rest are shown in black.

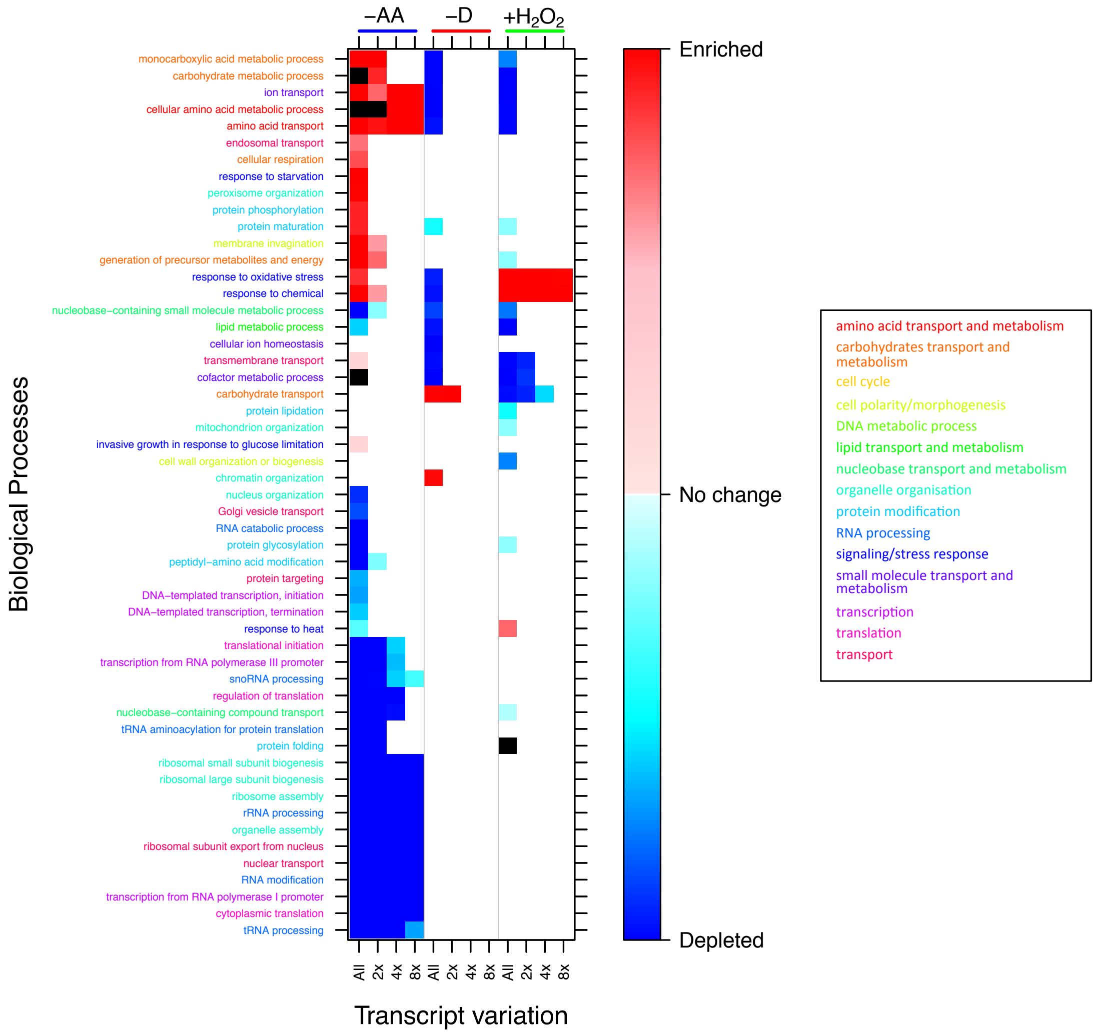

S26 Fig. Biological processes affected by stress response. GO Slim terms are that are overrepresented in the set significantly-enriched transcripts (red), the set of significantly-depleted transcripts (blue), or in both sets (black). Terms are grouped in 15 categories, and their name coloured accordingly.

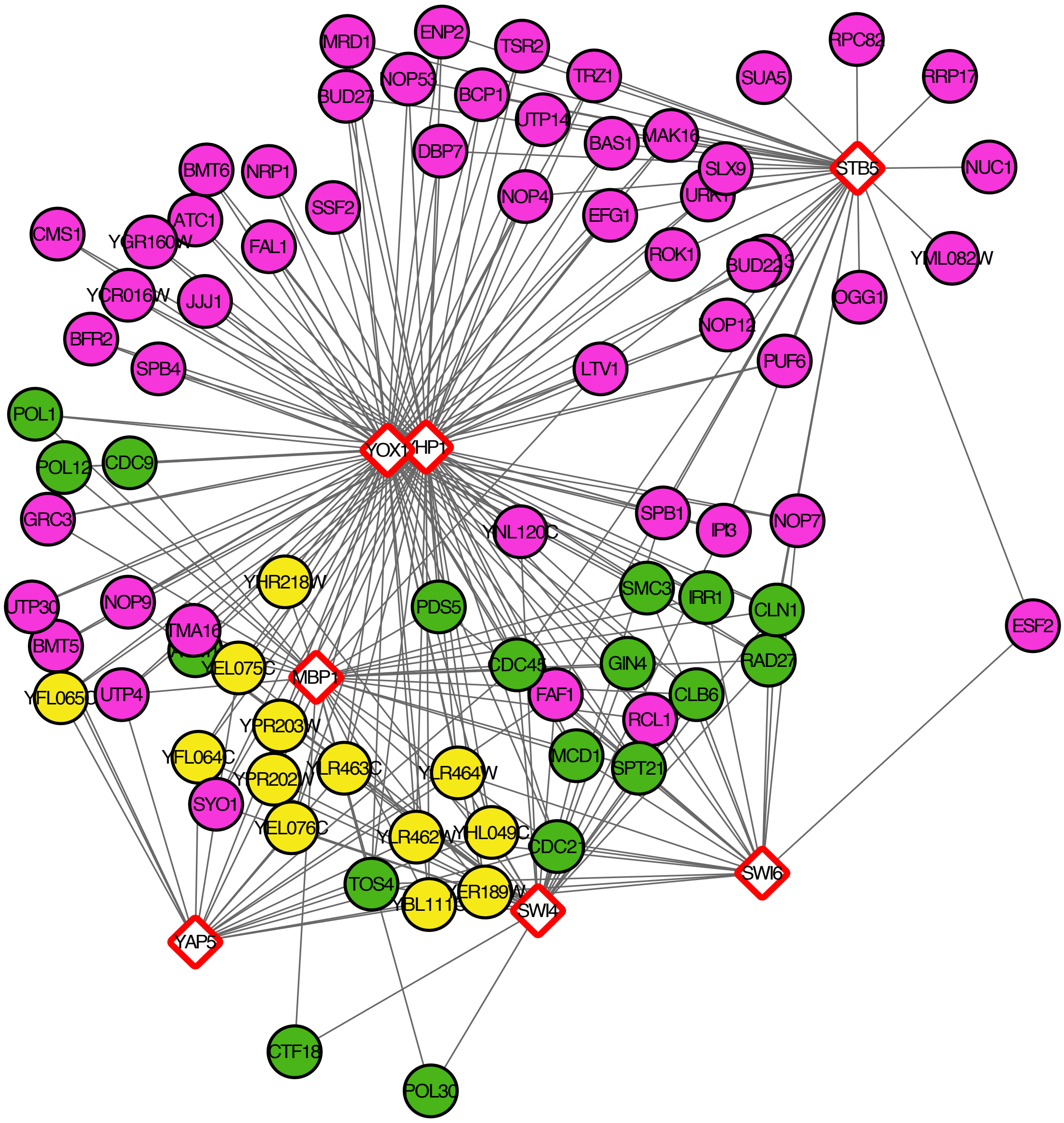

S27 Fig. Use of BTRs for functional annotation. Network of transcription factors associated with BTRs 60 (pink), 263 (yellow), and 330 (green); transcription factors shown as diamonds.
